# Molecular Response to Combined Molecular- and External Radiotherapy in Head and Neck Squamous Cell Carcinoma (HNSCC)

**DOI:** 10.3390/cancers13225595

**Published:** 2021-11-09

**Authors:** Treewut Rassamegevanon, Louis Feindt, Lydia Koi, Johannes Müller, Robert Freudenberg, Steffen Löck, Wiebke Sihver, Enes Çevik, Ariane Christel Kühn, Cläre von Neubeck, Annett Linge, Hans-Jürgen Pietzsch, Jörg Kotzerke, Michael Baumann, Mechthild Krause, Antje Dietrich

**Affiliations:** 1 German Cancer Consortium (DKTK), Partner Site Dresden, and German Cancer Research Center (DKFZ), 69192 Heidelberg, Germany; Treewut.Rassamegevanon@uniklinikum-dresden.de (T.R.); Steffen.Loeck@OncoRay.de (S.L.); Claere.VonNeubeck@uk-essen.de (C.v.N.); Annett.Linge@uniklinikum-dresden.de (A.L.); Mechthild.Krause@uniklinikum-dresden.de (M.K.); 2 German Cancer Research Center (DKFZ), 69192 Heidelberg, Germany; Michael.Baumann@dkfz.de; 3 OncoRay—National Center for Radiation Research in Oncology, Faculty of Medicine and University Hospital Carl Gustav Carus, Technische Universität Dresden, Helmholtz-Zentrum Dresden-Rossendorf, 01307 Dresden, Germany; Louis.Feindt@uniklinikum-dresden.de (L.F.); Lydia.Koi@uniklinikum-dresden.de (L.K.); Johannes.Mueller@uniklinikum-dresden.de (J.M.); mcevik15@ku.edu.tr (E.Ç.); ariane_christel.kuehn@mailbox.tu-dresden.de (A.C.K.); 4 Department of Radiotherapy and Radiation Oncology, Faculty of Medicine and University Hospital Carl Gustav Carus, Technische Universität Dresden, 01307 Dresden, Germany; 5 Helmholtz-Zentrum Dresden-Rossendorf, Institute of Radiooncology—OncoRay, 01328 Dresden, Germany; 6 Department of Nuclear Medicine, Faculty of Medicine and University Hospital Carl Gustav Carus, Technische Universität Dresden, 01307 Dresden, Germany; Robert.Freudenberg@uniklinikum-dresden.de (R.F.); Joerg.Kotzerke@uniklinikum-dresden.de (J.K.); 7 Helmholtz-Zentrum Dresden-Rossendorf, Institute of Radiopharmaceutical Cancer Research, 01328 Dresden, Germany; w.sihver@hzdr.de (W.S.); h.j.pietzsch@hzdr.de (H.-J.P.); 8 School of Medicine, Koç University, Istanbul 34450, Turkey; 9 B CUBE—Center for Molecular Bioengineering, Technische Universität Dresden, 01307 Dresden, Germany; 10 Department of Particle Therapy, University Hospital Essen, University of Duisburg-Essen, 45147 Essen, Germany; 11 National Center for Tumor Diseases (NCT), Partner Site Dresden, 01307 Dresden, Germany

**Keywords:** molecular targeted radiotherapy, DNA damage response, Cetuximab, cell death induction, external beam radiotherapy

## Abstract

**Simple Summary:**

Our previous preclinical trial in a head and neck squamous cell carcinoma (HNSCC) xenograft model showed a high potential for the improvement of curative treatment outcome upon the combination treatment of a radiolabeled (Yttrium-90) anti-EGFR antibody (Cetuximab) and external radiotherapy. We aim to elucidate the molecular response of HNSCC tumors upon this combination. Here, we show that the combination treatment leads to an increasing number and complexity of DNA double strand breaks. The upregulation of p21^cip1/waf1^ expression and cleaved caspase-3 suggest a blockage of cell cycle transition and an induction of programmed cell death. Collectively, a complex interplay between molecular mechanisms involved in cell death induction, cell cycle arrest, and DNA double strand break repair accounts for the beneficial potential using Yttrium-90-Cetuximab in combination with external radiotherapy.

**Abstract:**

Combination treatment of molecular targeted and external radiotherapy is a promising strategy and was shown to improve local tumor control in a HNSCC xenograft model. To enhance the therapeutic value of this approach, this study investigated the underlying molecular response. Subcutaneous HNSCC FaDu_DD_ xenografts were treated with single or combination therapy (X-ray: 0, 2, 4 Gy; anti-EGFR antibody (Cetuximab) (un-)labeled with Yttrium-90 (^90^Y)). Tumors were excised 24 h post respective treatment. Residual DNA double strand breaks (DSB), mRNA expression of DNA damage response related genes, immunoblotting, tumor histology, and immunohistological staining were analyzed. An increase in number and complexity of residual DNA DSB was observed in FaDu_DD_ tumors exposed to the combination treatment of external irradiation and ^90^Y-Cetuximab relative to controls. The increase was observed in a low oxygenated area, suggesting the expansion of DNA DSB damages. Upregulation of genes encoding p21^cip1/waf1^ (*CDKN1A*) and GADD45α (*GADD45A*) was determined in the combination treatment group, and immunoblotting as well as immunohistochemistry confirmed the upregulation of p21^cip1/waf1^. The increase in residual γH2AX foci leads to the blockage of cell cycle transition and subsequently to cell death, which could be observed in the upregulation of p21^cip1/waf1^ expression and an elevated number of cleaved caspase-3 positive cells. Overall, a complex interplay between DNA damage repair and programmed cell death accounts for the potential benefit of the combination therapy using ^90^Y-Cetuximab and external radiotherapy.

## 1. Introduction

HNSCC is the most common malignant disease arising in the oral cavity, oropharynx, hypopharynx, and larynx. It is considered as the sixth most common cancer worldwide with approximately 900,000 newly diagnosed cases in 2020 [1]. Radiotherapy with curative intent for HNSCC remains challenging as a subset of HNSCC patients are radioresistant [2]. In molecular targeted radiotherapy, molecules coupled with tumor-associated antigen-binding components are systemically administered, allowing targeted internal irradiation to primary cancers as well as distant metastases [3]. By combining the precise and conformal dose delivery to the primary tumor via external beam radiotherapy with molecular targeted radiotherapy, an increased tumor dose and potential targeting of metastases can be achieved, improving treatment outcome in HNSCC [4].

Epidermal growth factor receptor (EGFR), a transmembrane tyrosine kinase receptor, is involved in crucial cellular processes e.g., cell proliferation, differentiation, survival, and migration [5]. Overexpression of EGFR has been associated with an unfavorable treatment outcome in several tumor entities including HNSCC. Based on this, EGFR has been proposed as a potential target for molecular targeted radiotherapy [6]. Cetuximab, a monoclonal antibody targeting EGFR conjugated with radionuclides showed a therapeutic efficacy in a broad set of tumor types as monotherapy or combination therapy with chemo- or radiotherapy [4,7,8,9,10,11,12,13,14]. 

Yttrium-90 (^90^Y), a radioactive isotope of Yttrium, is a β-emitter with a half-life of 64.1 h and a maximum radiant energy of 2.27 MeV. It can affect water equivalent tissues within a range up to 12 mm, leading to the production of crossfire effect [15]. Therapeutic potential of ^90^Y-conjugated antibodies against EGFR as a combination treatment with external radiotherapy for HNSCC cells has been, therefore, investigated. Preclinical studies showed an enhanced cytotoxic effect upon the combined treatment, i.e., increased number and complexity of DNA double strand breaks (DSB), elevated cell death, and reduced proliferative capacity [14,16,17]. Finally, our previous preclinical trials in HNSCC tumor xenograft models [11,12] demonstrated that the combined treatment with external and molecular targeted radiotherapy using ^90^Y-Cetuximab substantially enhanced tumor control probability and reduced radiation dose to control 50% of tumors (TCD_50_) relative to the combination with unlabeled Cetuximab or external radiotherapy alone. Despite the improved efficacy of the combined treatment in tumor control, the underlying molecular effects supporting its clinical use remain elusive in preclinical experiments.

This study aims to elucidate the underlying molecular response induced by the dual therapy of ^90^Y-Cetuximab and external irradiation, leading to the effective tumor control shown in our previous preclinical trials [11,12]. Biological effects of exposure to radiation are primarily attributed to DNA DSB generation and cell death induction. Hence, we investigated residual γH2AX foci (DNA DSB marker) determined at 24 h post irradiation, mRNA expression of genes involved in DNA damage response (DDR), and the activation of programmed cell death in a HNSCC tumor xenograft model, namely FaDu_DD_. This study was designed and performed in parallel to our previously published study [12].

## 2. Materials and Methods

### 2.1. Animal Experiments

Ninety-four NMRI (nu/nu) mice (7–14 weeks old male and female, weighing 25–45 g) were acquired from OncoRay National Center for Radiation Research in Oncology (Dresden, Germany). Animals were housed in a group of 8–10 animals in a conventional EU Standard Type III cage with a filter top and a wire lid under controlled environment: temperature (25–27 °C), humidity (50–60%), and light–dark cycle (12:12 h). Animals had access to autoclaved dry feed and water ad libitum. The pathogen-free animal facility (OncoRay National Center for Radiation Research in Oncology, Dresden, Germany) and the experiments were approved by the regulatory authorities (AZ: DD24-5131/207/15) in accordance with the European Parliament and Council (EU Directive 2010/63/EU) on the protection of animals used for scientific purposes, the German animal welfare regulations, and the local animal ethics committee. The experiments were performed and reported in adherence to ARRIVE guidelines [18]. FaDu_DD_, an established human HNSCC tumor model, was investigated in this study. The tumor characteristics were reported elsewhere [19,20]. The xeno-transplantation of tumors was described previously [21]. In brief, animals were whole-body irradiated with 4 Gy (X-rays, Maxishot 200 Y.TU/320-D03, Yxlon International, Hamburg, Germany; 200 kV, 20 mA; 0.5 mm Cu filter; dose rate 1 Gy/min) prior to subcutaneous transplantation of FaDu_DD_ tumor pieces (~ 2 × 2 mm) from a source tumor into the right hind-leg of anesthetized animals using Ketamine (Ketamin 500 Curamed^®^, CuraMed Pharma, Karlsruhe, Germany, 120 mg/kg) and Xylazine (Rompun^®^, Bayer Healthcare, Leverkusen, Germany, 16 mg/kg). Histology and volume doubling time were assessed in control animals (*n* = 4) and microsatellite analysis of the source tumors was carried out to confirm the tumor identity. The animals were excluded from the experiment if they showed no tumor growth, second nodules in proximity to the transplantation site, or health issues subject to the exclusion criteria according to the German animal welfare regulations (*n* = 15).

**Figure 1:**
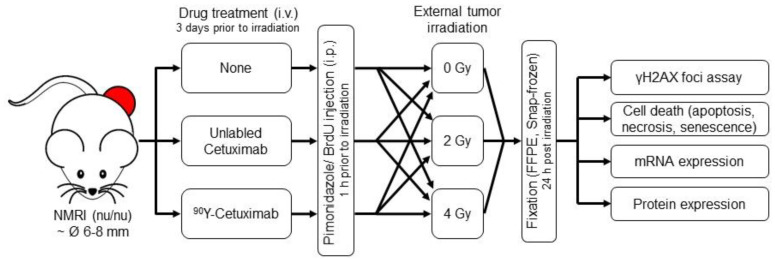
Overview of the animal experiment. Schematic illustration of the animal experiment. The experiment was carried out in two cohorts in parallel to the preclinical trial testing treatment efficiency published in [12]. Upon reaching a tumor diameter of 6–8 mm, animals were allocated to nine different treatment arms. Drugs were injected intravenously 3 days prior to irradiation. Pimonidazole (hypoxic marker) and bromodeoxyuridine (BrdU; proliferation marker) were administered intraperitoneally 1 h before tumors were locally exposed to external irradiation with 0, 2 or 4 Gy. Tumors were fixed 24 h post irradiation and the fixed samples were used for further molecular analysis. The total number of animals in each treatment arm is shown in Table S1.

Animals bearing tumor with a diameter of 6–8 mm were allocated into nine treatment arms i.e., untreated, monotherapy of external tumor irradiation with 0, 2 or 4 Gy, ^90^Y labeled or unlabeled Cetuximab (Erbitux^®^; Merck KGaA, Darmstadt, Germany), or the combination therapy. The overview of experimental design is illustrated in Figure 1. Randomization of animals could be achieved as FaDu_DD_ tumors reached the expected size asynchronously [19,22]. ^90^Y (2.8 MBq) labeled or unlabeled Cetuximab (13 µg) was administered intravenously into the tail vein of animals 3 days prior to external tumor irradiation. Cetuximab was acquired from the local hospital pharmacy. The radiolabeling procedure of Cetuximab with ^90^Y is described in detail elsewhere [12,17]. Due to the local radioprotection regulations, investigators could not be blinded as to whether the animal was injected with ^90^Y labeled or unlabeled Cetuximab. One hour before external radiation exposure, animals were intraperitoneally injected with pimonidazole (hypoxia marker; Natural Pharmacia International, Burlington, MA, USA; 0.1 mg/g animal body weight) and bromodeoxyuridine (BrdU, proliferation marker; SERVA electrophoresis, Heidelberg, Germany; 3.75 mg). For the single dose external tumor irradiation under ambient blood flow condition, animals were immobilized in plastic tubes, which were placed on an acrylic plate, and a custom-made clipper was used to position the tumor-bearing leg within the irradiation field. Animals were sacrificed 24 h post external tumor irradiation; tumors were excised and cut in half. One half of the tumor was snap-frozen in liquid nitrogen and the other half was formalin-fixed and embedded in paraffin (FFPE). 

Due to the residual radioactivity, the formalin fixation and the dehydration in a grade ethanol series were conducted in a radiation protection area and the tissue processing duration was extended to approx. 24 h for each step. In total, 75 animals were allocated to the respective treatment arms as shown in Table S1. The experiment was designed and conducted in parallel to the previously published study on the therapeutic efficiency of the combined treatment between ^90^Y-Cetuximab and external beam radiation with tumor control as curative endpoint [12].

### 2.2. Radioactivity Measurement of ^90^Y in Tumors and Organs

Radioactivity in tumors, spleen, liver, and kidney of animals treated with ^90^Y-labeled Cetuximab were measured at the day of sacrifice by using a calibrated automated gamma counter (Packard Cobra II, Canberra, IL, USA). The relative uptake in organs was calculated as the ratio of the decay corrected organ activity and the injected activity.

### 2.3. Immunofluorescence, Immunohistochemistry and Histological Staining

All stainings were carried out on FFPE samples with a section thickness of 3 µm. Standard hematoxylin and eosin (H&E) staining was performed on tumor sections. Two consecutive sections of tumors were stained for pimonidazole/BrdU (Immunohistochemistry: IHC) using ARK^TM^ Kit (Animal Research Kit; Agilent Technologies Deutschland, Hamburg, Germany) and γH2AX (Immunofluorescence: IF) using Alexa Flour^TM^ 488 Tyramide Signal Amplification Kit (Invitrogen, Darmstadt, Germany) [23]. The tumor sections were probed for p21^cip1/waf1^ and cleaved caspase-3. IHC and IF staining were counterstained with hematoxylin and 4′,6-diamidino-2-phenylindole (DAPI), respectively. The list of antibodies and kits used in this study is provided in Table S2.

### 2.4. Determination of RNA Expression

The isolation of total RNA from frozen tumor samples was carried out using RNeasy micro kits (#74004, Qiagen, Hilden, Germany) and the total RNA concentration was determined using Qubit^TM^ RNA HS Assay Kit (#Q32852, Invitrogen, Darmstadt, Germany). A microarray with custom-made codesets for 223 DDR genes and 7 housekeeping genes was purchased from the manufacturers (Nanostring Technologies, Seattle, WA, USA; Integrated DNA Technologies, Leuven, Belgium) and mRNA expression was determined with nCounter^TM^ (Nanostring Technologies, Seattle, WA, USA) according to the manufacturer’s protocol. The list of custom-made gene sets is provided in the Table S3. To validate mRNA expression assessed by nCounter^TM^, real-time qRT-PCR of a set of 18 genes was carried out. RNA was reverse-transcribed to cDNA. PCR reaction mix was prepared by mixing cDNA, TaqMan^®^ Master Mix (#4444556, Applied Biosystems, Waltham, MA, USA), TaqMan^®^ assays (Applied Biosystems, Waltham, MA, USA), and nuclease-Free water (#129114, Qiagen, Hilden, Germany). Real-time qRT-PCR was performed on 96-well plates in StepOnePlus^TM^ Real-Time PCR System (#4376600, Applied Biosystems, Waltham, MA, USA) with the following run conditions: 2 min at 50 °C, 2 min at 95 °C, and 40 cycles of 1 s at 95 °C and 20 s 60 °C. The list of genes and primers is provided in the Table S4. Genes that showed significant up- or downregulation upon treatments were used for a functional annotation analysis and protein network analysis using an online tool (DAVID Bioinformatics Resources 6.8 [24]). KEGG database [25] was used as the reference.

### 2.5. Immunoblotting

Proteins were isolated from FFPE tumor samples. Depending on the size of the tumors, several sections with a thickness of 3 µm (30–50 sections) were prepared. FFPE tumor sections were deparaffinized in xylene, rehydrated with a graded series of ethanol, and briefly air-dried under a fume hood. Tissue lysis buffer (0.1 M Tris-HCl, pH 8.0, 0.1 M DTT, 4% (*w*/*v*) SDS) of 50–100 µL was added and incubated while agitating at 100 °C for 20 min and 90 °C for 100 min. The whole tissue lysate was centrifuged and protein concentration was determined by the tryptophan fluorescence assay, as previously described [26]. SDS PAGE (12% SDS gel concentration) was performed and resolved proteins were transferred to a nitrocellulose membrane using a wet-blotting system (Mini Trans-Blot^®^ Cell, Bio-Rad Laboratories, Feldkirchen, Germany). Membranes were probed for p21^cip1/waf1^, GADD45α and β-tubulin. The immunoblotting was documented using Vilber FsusionFX system (Vilber Lourmat Deutschland, Eberhardzell, Germany) or ChemiDoc MP Imaging system (Bio-Rad Laboratories, Feldkirchen, Germany). The densitometry of the blots was determined with a Fiji ImageJ built-in function—Gel analysis (Version 1.53k) [27]. 

### 2.6. Image Acquisition and Analysis

H&E images were acquired using a wide-field slide scanner (Axioscan Z1; Carl Zeiss, Jena, Germany) with a 10× objective (Plan-Apochromat 10×/0.45, Carl Zeiss, Jena, Germany). Whole section scanning of IHC images and IF images were carried out with a 10× objective (Plan-Apochromat 10×/0.45, Carl Zeiss, Jena, Germany) using wide-field fluorescence microscopes (Axio Imager Z2 or Axio Imager M1; Carl Zeiss, Jena, Germany) equipped with dual cameras (digital color camera: Axiocam MRc, and monochrome camera: AxioCam MRm; Carl Zeiss, Jena, Germany). For acquisition of IF images stained for γH2AX, Axio Imager M1 with a 40X objective (Plan-Apochromat 40×/0.45; Carl Zeiss, Jena, Germany) was used. Microscopes were controlled by ZEN software (Version 3.1; Carl Zeiss, Jena, Germany) or AxioVision (Version 4.9; Carl Zeiss, Jena, Germany). During IF acquisition, exposure time was kept constant for an entire staining batch. 

The in vivo γH2AX foci assay was carried out as previously described [23,28]. Briefly, in each tumor section, seven to fifteen regions of interest (ROI) with a single, pimonidazole negative blood vessel surrounded by BrdU positive cells were selected for the following acquisition of γH2AX. For each ROI, focus z-stack images (17 images with a focus interval of 0.25 µm) covering a distance of 250 µm from the nearest perfused vessel were acquired. An extended depth focus image using the maximum projection method was generated from the z-stack images for the further image analysis. Within a ROI, the distance to the nearest perfused vessel was classified into three categories i.e., <50 µm, 50–100 µm, and >100 µm. Within 50 µm distance from the nearest perfused vessel, pimonidazole remained negative for every ROI [28]. Analyzable cells within the three distance categories were numbered and five cells per category were randomly selected for the manual foci enumeration. Gamma H2AX foci size and mutual distance among foci was determined in cells within the distance of up to ~100 µm from the nearest perfused vessels using an extended version of a Fiji ImageJ compatible macro (FociCounter; https://github.com/jo-mueller/FociCounter, accessed on 12 March 2021) [29] developed for this study. In brief, cells were segmented [30] and foci were detected using Fiji’s maximum finder method. The script then counts the number of foci per cell, size of each focus and the mutual distance between all foci within a given nucleus. The latter is defined as the median Euclidian distance between all present foci. Corrected foci (cfoci) were calculated as described previously [31]. Briefly, the mean of the nucleus area was multiplied by the quotient of foci number and area of an individual nucleus. The means of nucleus area were calculated in a distance category- and tumor-specific manner. Tumors which were BrdU and Pimonidazole negative were excluded from the analysis (*n* = 9). For analysis of p21^cip1/waf1^, cleaved caspase-3 positive cells, and necrotic fraction, QuPath, an open-source bioimage analysis software [32], was used. For the evaluation of necrotic fraction, several annotations of necrotic fraction, tumor fraction, and stroma on each individual tumor were performed manually as training data for the automatic segmentation using the built-in random forests classifier, which predicts the percentages of each component. Cleaved caspase-3 and p21^cip1/waf1^ positive cells were segmented and classified into three categories based on the fluorescence intensity. The thresholding method for the classification of fluorescence intensity is described in the Supplementary Materials. The number of positive cells was normalized with total cell number. 

### 2.7. Statistical Analysis

Linear mixed-effects model or analysis of variance (ANOVA) were applied. For multiple comparison and post-hoc test, Sidak’s correction was carried out. Statistical analyses were conducted with SPSS Software (Version 27, IBM Deutschland, Ehningen, Germany). Data visualization was performed using GraphPad Prism (Version 7, GraphPad Software, San Diego, CA, USA) and Rstudio [33] with ggplot2 package. Data of mRNA expression from nanoString^TM^ were analyzed using nSolver^TM^ software (Version 4.0; Nanostring Technologies, Seattle, WA, USA).

## 3. Results

**Figure 2:**
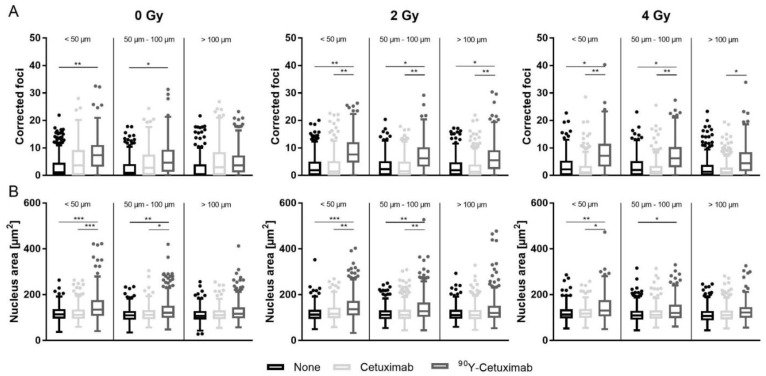
Analysis of DNA damage and cell nucleus area determined by γH2AX foci assay upon monotherapy or combination therapy. Corrected foci (cfoci) and nucleus area of FaDu_DD_ xenograft tumors treated with monotherapy or combination therapy of external tumor irradiation (0, 2, or 4 Gy) plus Cetuximab or ^90^Y-Cetuximab. FFPE tumors were stained for γH2AX and counterstained with DAPI. The range for γH2AX foci enumeration was classified into three categories based on the distance from the nearest perfused vessel (<50 µm, 50–100 µm, and >100 µm). Five analyzable nulcei per distance category were randomly selected for manual assessment of γH2AX foci counts (**A**) and nucleus size measurement (**B**). cfoci were calculated and a linear mixed-effects model was performed on square root transformed cfoci and log transformed nucleus area. (*: *p* < 0.05, **: *p* < 0.01, ***: *p*< 0.001).

The total uptake of radionuclide observed in FaDu_DD_ xenograft tumors, livers, kidneys and spleens of animals treated with ^90^Y-Cetuximab is shown in Figure S1. To investigate the effect of ^90^Y-Cetuximab inflicting DNA DSB damages, residual γH2AX foci were determined manually in nuclei located within three distance categories based on the distance from the nearest perfused vessel (<50 µm, 50–100 µm, and >100 µm, respectively). The outputs of descriptive statistics and linear mixed-effects model are shown in Tables S5 and S6. On average, 350 cell nuclei/distance categories were analyzed for γH2AX foci in each treatment arm. Injection of ^90^Y-Cetuximab without external tumor irradiation caused a significant increase in residual γH2AX foci up to 100 µm compared to unlabeled Cetuximab or untreated control (Figure 2A). Upon the combination treatment with ^90^Y-Cetuximab and external beam radiation, a significant increase in residual γH2AX foci, depending on the distance to the nearest perfused vessel, could be observed, while this increment could not be detected in tumors treated with the combination of external irradiation with unlabeled Cetuximab or radiation alone. A reduction of residual γH2AX foci in a distance-dependent manner could be found solely in ^90^Y-Cetuximab-treated tumors, indicating that a DNA DSB infliction of ^90^Y-Cetuximab decreased gradually with distance to the nearest perfused vessel (Figure S2). 

In the second analysis, γH2AX foci were automatically analyzed using an updated version of the FociCounter in the cell nuclei located within a distance of approx. 0–100 µm from the nearest perfused blood vessel. Statistical analysis with a linear mixed-effects model of corrected residual γH2AX foci analyzed by the algorithm showed a similar outcome as the manual evaluation (Figure S3). The mutual distance among residual γH2AX foci in the individual cell nuclei became narrow in tumors exposed to ^90^Y-Cetuximab. Its combination with external beam radiation generated more cells with higher residual γH2AX cfoci and smaller distance among foci compared to the combination of external tumor irradiation with unlabeled Cetuximab or untreated control (Figure S4). This suggests complex residual DNA DSB damages introduced by the combination treatment of external irradiation and ^90^Y-Cetuximab. A significant increase in cell nucleus area, which was located in the distance to the nearest perfused vessel up to 100 µm, upon ^90^Y-Cetuximab treatment could be determined compared to monotherapy of unlabeled Cetuximab or control, independent of irradiation (Figure 2B). Overall, combination treatment of ^90^Y-Cetuximab and external irradiation increased the presence of residual DNA DSB and induced complex DNA DSB.

**Figure 3:**
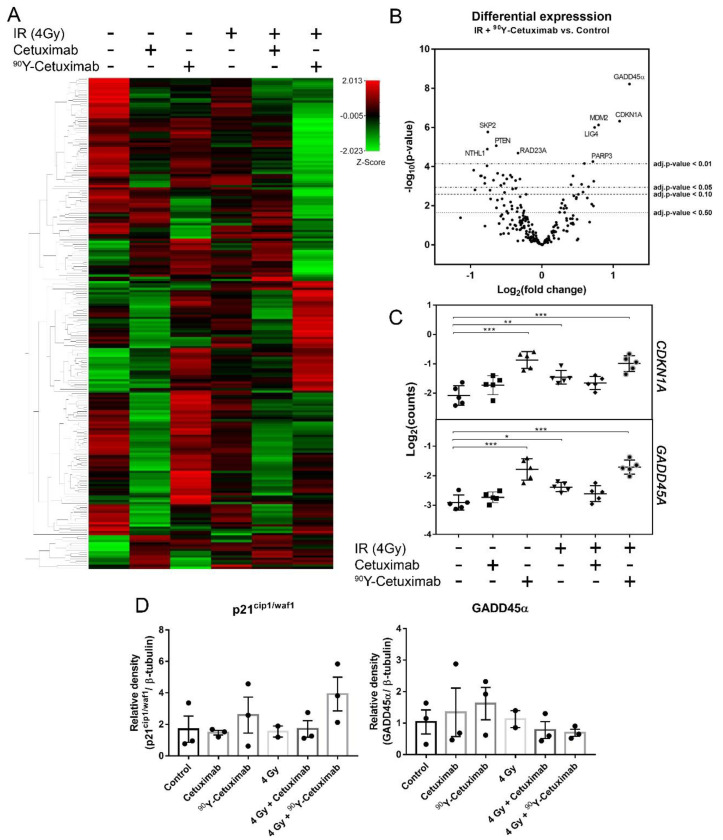
mRNA and protein expression of DDR genes upon mono- and combination treatment with external irradiation and ^90^Y-(un-)labeled Cetuximab. Heat map of mRNA expression of DDR related genes determined by nCounter^TM^ (*n* = 5) (**A**). Differential mRNA expression of the combination treatment using external irradiation (4 Gy) with ^90^Y-Cetuximab relative to control (**B**). Log_2_Counts of two candidate genes: *CDKN1A* and *GADD45A* under different treatment arms (**C**). Solid lines and error bars denote mean and standard deviation, respectively. One-way ANOVA followed by post-hoc test with Sidak’s correction for the comparison between the treatment groups and control was performed (*: *p* < 0.05, **: *p* < 0.01, ***: *p* < 0.001). Protein expression of *CDKN1A* encoding protein, p21^cip1/waf1^, and GADD45α assessed by immunoblotting (*n* = 3) (**D**). Bars and error bars represent mean and standard error of mean, respectively.

To study the molecular responses of FaDu_DD_ tumors upon the combination treatment with ^90^Y-Cetuximab and external irradiation, mRNA expression of DDR genes was assessed on RNA isolated from cryopreserved tumors using the nanoString^TM^ platform. The outcome of nanoString^TM^ was validated by real time qRT-PCR on 18 selected genes. Expression determined by nanoString^TM^ showed a good degree of correlation with the result from real time qRT-PCR (r^2^ = 0.7276; Figure S5). The heatmap shows a distinct mRNA expression pattern among the treatment arms, except for the group with unlabeled Cetuximab single treatment and its combination with external irradiation, where a similar pattern of expression could be observed (Figure 3A). According to the differential expression analysis, the comparison between untreated control and tumors treated with ^90^Y-Cetuximab and external irradiation (Figure 3B) demonstrated that several genes were significantly upregulated (*GADD45A, CDKN1A*, *MDM2* and *LIG4*) or downregulated (*PTEN, RAD23A*, *RPA1, RAD51C* and *PARP1*). The top 20 genes that were significantly altered in each treatment arm compared to untreated tumors are shown in Table S7. Functional annotation analysis of genes that are significantly upregulated showed a significant enrichment in p53 signaling pathway as well as cell cycle upon monotherapy of ^90^Y-Cetixumab (Table S8). Tumors treated with the combination of ^90^Y-Cetuximab and external irradiation exhibited a significant gene enrichment in nucleotide excision repair and p53 signaling pathway for upregulated genes, and base excision repair, cell cycle and Fanconi anemia pathway for downregulated genes (Table S9).

As proteins encoded by *GADD45A* (GADD45α) and *CDKN1A* (p21^cip1/waf1^) are essential components in cell cycle arrest and programmed cell death mechanisms, their expression was further investigated. The comparison among the treatment arms demonstrated a significant increase in *GADD45A* and *CDKN1A* mRNA expression in tumors treated with ^90^Y-Cetuximab (Figure 3C). Subsequently, protein expression assessed by immunoblotting of GADD45α and p21^cip1/waf1^ was performed. p21^cip1/waf1^ expression was in line with its mRNA expression. In contrast, protein level of GADD45α remained unaltered in all the treatment arms compared to control (Figure 3D). The original images of immunoblotting are provided in Figure S6.

**Figure 4:**
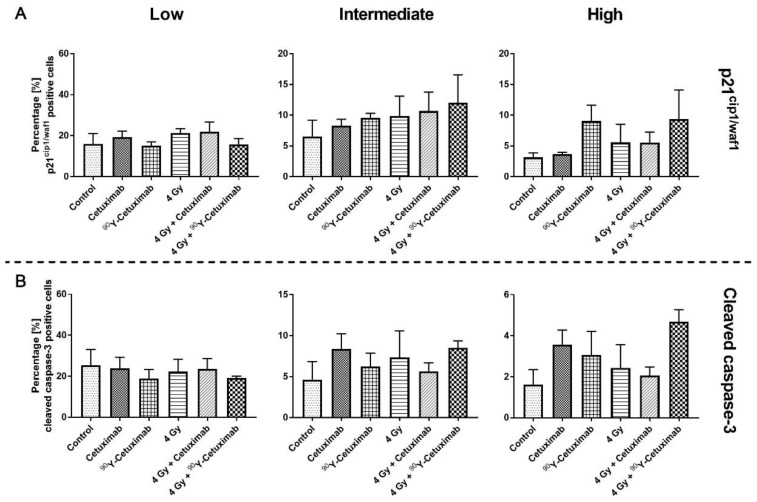
Analysis of p21^cip1/waf1^ and cleaved caspase-3 positive cells. Whole tumor section scans of immunofluorescence staining were acquired. p21^cip1/waf1^ (*n* = 3, (**A**)) and cleaved caspase-3 (*n* = 4, (**B**)) positive cells were determined. Tumor region was annotated and cells within annotation were automatically segmented. Intensity of fluorescent signal of the two markers was classified into three categories i.e., low, intermediate, and high based on the distribution of mean intensity (see Supplementary Method). Percentage of positive cells and total cell number were determined. Bars and error bars represent mean and standard error of mean, respectively.

To observe cell death and the activation of cell death mechanism in FaDu_DD_ tumors induced in each treatment arm, tumors were immunofluorescence stained for cleaved caspase-3 and p21^cip1/waf1^, which are markers for apoptosis and cell cycle arrest, respectively. (Figures S7 and S8). In addition, H&E staining was carried out to evaluated necrotic fraction. The histological analysis of necrosis showed insignificant changes in the necrotic fraction among the treatment arms (data not shown). Cleaved caspase-3 as well as p21^cip1/waf1^ positive cells could be observed in all conditions. A higher number of p21^cip1/waf1^ positive cells with high intensity were observed in tumors treated with ^90^Y-Cetuximab (monotherapy and combined with external irradiation) compared to control or unlabeled Cetuximab (Figure 4A), which is in line with the immunoblotting outcome. Similarly, the highest number of cleaved caspase-3 positive cells with high intensity can be seen in the combination treatment of ^90^Y-Cetuximab with external irradiation of 4 Gy (Figure 4B). This suggests that the combination treatment, where the radiation dose is higher relative to other treatment arms, enhanced cell death induction as well as cell cycle arrest.

## 4. Discussion

In previous preclinical trials, we could demonstrate that the combination treatment of ^90^Y-Cetuximab and a single radiation dose [11] or fractionated external irradiation [12] substantially increased the control rate and reduced TCD_50_ of FaDu_DD_ xenograft tumors. In the present study, we used tumor xenograft specimens from an experiment that was conducted in parallel to the study on therapeutic efficacy of the combination treatment [12]. The molecular response upon the combined molecular targeted and external beam radiotherapy was investigated via residual DNA DSB, mRNA expression of DDR genes and the corresponding protein expression of the two candidate genes that showed the highest upregulation upon the combination treatment, and the activation of cell death induction. Overall, the results reflect the outcome of the tumor control experiment [12], suggesting more extensive and complex residual DNA damages, and a pronounced induction of cell cycle arrest and programmed cell death, in particular apoptosis, upon combination of ^90^Y-Cetuximab with external irradiation. 

One of the major mechanisms of radiation-induced cellular cytotoxicity is the generation of reactive oxygen species. Their reduction in hypoxic tumor microenvironment contributes to enhanced tumor radioresistance [34]. From blood vessels, the typical oxygen diffusion distance is estimated to be 100–150 µm with a rapid drop in oxygen concentration along the distance [35,36]. Although Cetuximab does not penetrate far into the tissue with the dose applied in this study [12], the induction of DNA damage within a range up to 12 mm via ^90^Y is estimated due to the crossfire effect [15]. We showed that the combination treatment of ^90^Y-Cetuximab and external beam irradiation enhanced the numbers of residual DNA DSB, which will eventually trigger cell death, in cell nuclei located beyond 100 µm distance away from the nearest perfused vessels. This effect was found to be statistically significant in tumors treated with the combination of ^90^Y-Cetuximab and external beam irradiation of 2 Gy, and a similar trend can be seen when the combined treatment of ^90^Y-Cetuximab and external beam irradiation of 4 Gy was applied (Tables S5 and S6). This suggests that the combination treatment could extend damages to cells located in an area with low oxygen concentration. In addition, residual γH2AX foci detected in tumors treated with ^90^Y-Cetuximab and external tumor irradiation were densely localized in contrast to tumors treated with monotherapy or unlabeled Cetuximab with external irradiation, implying more complex, persistent DNA damages induced by the combined molecular and external radiation treatment. Studies showed that chemo- or radiotherapy induced the nuclear internalization of radionuclide-labelled and unlabeled EGFR [13,14,37,38], increasing absorbed dose deposited within the tumor [39]. This supports the enhanced DNA damage complexity upon the combined treatment shown in this study. 

DDR genes are frequently altered upon exposure to radiation [9,40]. FaDu_DD_ tumor xenografts treated with ^90^Y-Cetuximab in combination with external irradiation showed the most pronounced transcriptional alteration of DDR genes in contrast to the other treatment arms. The functional annotation analysis of downregulated genes exhibited an enrichment in DNA damage response pathways i.e., base excision repair, Fanconi anemia, cell cycle progression, and nucleotide excision repair. These pathways are responsible for DNA single strand break repair, and their dysregulations could lead to a subsequent formation of DNA DSB [41]. Downregulated mRNA expression of *RAD51C* and *RPA1*, which encode key proteins responsible for DNA DSB repair via homologous recombination (HR), as well as *PARP1*, an essential regulator for single strand and double strand damage repair mechanism, were observed, whereas the mRNA of genes involved in non-homologous end joining (NHEJ) remained unaltered in the combined treatment group with ^90^Y-Cetuximab and external irradiation relative to control (Table S9). Studies showed essential roles of PARP1 in modulating DNA end resection [42] and recognizing DNA damages [43], facilitating DNA DSB repair via HR. Based on this, we hypothesize that the combination treatment of ^90^Y-Cetuximab and external radiotherapy impedes DNA DSB repair pathway via HR, causing the high number of residual γH2AX foci [44].

A microarray analysis of the syngeneic murine melanoma model B16F10 upon a dual treatment of carbon-ion external radiotherapy and ^131^I-Benzamide showed an enrichment of the genes *PARP3, MDM2, GADD45A* [9], which were also found to be upregulated in our study. The FaDu_DD_ model possesses a homozygous mutation of *TP53* [20] and *MDM2*, a central antagonist of p53. Since GADD45α, a crucial G2/M phase transition inhibitor, is primarily regulated by p53 [45], these might explain the unaltered GADD45α protein expression upon the combination treatment. In contrast, the induction of p21^cip1/waf1^ is associated with cellular response mechanisms to genotoxic stress, i.e., cell cycle arrest [46], apoptosis [47], senescence [48], and DNA damage repair [49], which can be mediated in p53-dependent and -independent manners [50]. The upregulation of p21^cip1/waf1^ could be observed on the mRNA level as well as protein level in the combined treatment group of ^90^Y-Cetuximab and external irradiation but not in monotherapy or the combination of external irradiation and unlabeled Cetuximab. Moreover, an increased expression of cleaved caspase-3 was identified. This indicates an increased radiotoxicity and an enhanced radiation-induced mitotic catastrophe upon the combination treatment of ^90^Y-Cetuximab and external irradiation, which could be explained by the dose effect.

In this study, we showed the high number and complexity of residual γH2AX foci, the alteration in DDR genes as well as the enhanced cellular cytotoxicity upon the combination treatment with ^90^Y-Cetuximab and external irradiation. These effects could be induced by the higher radiation dose applied in the combination treatment arm relative to other treatment arms. Initially, the administration of ^90^Y-Cetuximab generates DNA damages. The subsequent application of external beam radiotherapy introduces additional DNA damages and might complicate the pre-existing damages caused by ^90^Y-Cetuximab. This sequence of DNA damage induction could trigger cell cycle arrest and cellular cytotoxicity, leading to the high therapeutic efficacy of the combination treatment shown in the preclinical trail focusing on efficacy which was conducted in parallel [12].

In a phase 3 multicenter randomized trial, the dual therapy of Cetuximab and fractionated external beam radiotherapy in patients with advanced HNSCC led to prolonged 5-year overall survival compared to external radiotherapy alone [51,52]. Molecular effects of the combination therapy in HNSCC are contributed to by decreasing proliferation [53], inducing cell cycle arrest [54], increasing apoptosis [55], improving tumor reoxygenation [56,57], and impairing DDR [58]. Our study focused on DDR and cell death induction. No significant molecular effects induced by unlabeled Cetuximab or its combination with external irradiation could be identified. The results are consistent with our previous preclinical trials, where only a slight improvement in TCD_50_ was determined in the treatment arms with unlabeled Cetuximab combined with single-dose [11] or fractionated irradiation [12]. This could be explained by virtue of the dose of Cetuximab applied in our studies. As this dose is determined by the prescribed activity of ^90^Y, it is only a fraction of the Cetuximab dose used in other preclinical settings, in which the cellular and molecular effects of the combined treatment of Cetuximab with external irradiation were determined [53,59]. This suggests that clinical settings of the radioimmunotherapy, which often include the application of some cold dose prior to injection of radiolabeled compounds [60,61], would potentially enhance cellular cytotoxicity and, consequently, improve the treatment efficacy in a combined treatment strategy. 

The tumor and normal tissue uptake of ^90^Y-Cetuximab measured in our study was comparable to reports from other preclinical experiments [9,62]. A study of the distribution of ^89^Zr-Cetuximab in xenograft models of four different entities at 96 h post administration showed a range of uptake in tumors from 1 to 10%, depending on tumor models, whereas the uptake in normal tissue was similar in all the models [62]. Interestingly, the EGFR expression level of those models did not correlate with the positron emission tomography imaging (PET) signal acquired in vivo, suggesting complex mechanistic cues in the uptake of Cetuximab in tumors [62,63]. Regardless of the underlying mechanisms, a sufficient uptake of the radiolabeled compound is a prerequisite for the beneficial effect of a combined molecular and external radiotherapy [4,11]. Thus, a theranostic approach using ^86^Y-Cetuximab to monitor the uptake of the antibody in HNSCC tumors with PET prior to, or during the course of fractionated radiation treatment has to be introduced for clinical translation. This may allow the stratification of patients that would benefit from the combination treatment, facilitating personalized combination treatment [4,6,11,12].

## 5. Conclusions

Our study provides insights into the molecular response upon the combination treatment of ^90^Y-Cetuximab and external irradiation in a preclinical setting. The distribution of persistent DNA DSB damages to the area with low oxygen concentration, the enhancement of DNA DSB complexity, and the increased cellular cytotoxicity underline the efficacy of the dual treatment with ^90^Y-Cetuximab and external irradiation. This highlights the promising potential in the clinical translation of combined molecular targeted radiotherapy and external radiation treatment regimes.

## Data Availability

The dataset generated during the current study is available from the corresponding author on reasonable request. The data are not publicly available due to the institutional guidelines.

## Supplementary Materials

The following are available online at https://www.mdpi.com/article/10.3390/cancers13225595/s1, Supplementary method, Figure S1: In vivo biodistribution of ^90^Y-Cetuximab, Figure S2 Corrected residual γH2AX foci plotted dependent on the distance from the nearest perfused vessel, Figure S3: Corrected residual γH2AX foci determined by manual or FociCounter in nuclei located within the distance up to 100 µm from the nearest perfused vessel., Figure S4: Two-dimensional density plot of corrected γH2AX foci number and mutual distance among foci determined by FociCounter, Figure S5: Correlation analysis of data from nanoString^TM^ and real-time qRT-PCR, Figure S6: Full-length of immunoblotting images probed for GADD45α, p21^cip1/waf1^, and β-Tubulin (A) and densitometry of each lane (B), Figure S7: Representative images of immunofluorescence staining of p21^cip1/waf1^, Figure S8: Representative images of immunofluorescence staining of cleaved caspase-3, Table S1: Total number of animals eligible for the study, Table S2: List of antibodies, kits, and chemicals used in this study, Table S3: Customized gene set and the corresponding accession number for DNA damage response related mRNA expression analysis with nanoString^TM^, Table S4: Primer list for real-time qRT-PCR, Table S5: Summary of descriptive statistics from manual γH2AX foci determination, Table S6: Statistical output of corrected foci and nucleus area analyzed by a linear mixed-effects model based on the manual assessment of γH2AX foci, Table S7: Top 20 differentially expressed DNA damage response related genes in the treated groups relative to the untreated group determined by nSolver^TM^, Table S8: Functional annotation analysis of genes that significantly up- and downregulated upon monotherapy of ^90^Y-Cetuximab, Table S9: Functional annotation analysis of genes that significantly up- and downregulated upon the combination therapy of ^90^Y-Cetuximab and external tumor irradiation of 4 Gy.
