# Supplementary material for: Molecular Response to Combined Molecular- and External Radiotherapy in Head and Neck Squamous Cell Carcinoma (HNSCC)
Source: Cancers (Basel). 2021 Nov 9;13(22):5595. doi: 10.3390/cancers13225595 (PMC8615625; doi:10.3390/cancers13225595)
Supplement: Supplementary file 1 [file cancers-13-05595-s001.zip › cancers-1453843-supplementary.pdf]

Supplementary materials

Molecular response to combined molecular- and external radiotherapy in head and neck squamous cell carcinoma (HNSCC)

Treewut Rassamegevanon [1,2,3], Louis Feindt [3,4], Lydia Koi [3,4,5] Johannes Müller [3,5], Robert Freudenberg [6], Steffen Löck [1,2,3,4], Wiebke Sihver [7], Enes Cevik [3,5,8], Ariane Christel Kühn [3,9], Cläre von Neubeck [1,2,3,10], Annett Linge [1, 2, 3, 4, 11], Hans-Jürgen Pietzsch [7], Jörg Kotzerke [6], Michael Baumann [2, 3], Mechthild Krause [1,2,3,4,5,11], Antje Dietrich [1,2,3,*]

1 German Cancer Consortium (DKTK), Partner Site Dresden, and German Cancer Research Center (DKFZ), 69192, Heidelberg, Germany
2 German Cancer Research Center (DKFZ), 69192, Heidelberg, Germany
3 OncoRay—National Center for Radiation Research in Oncology, Faculty of Medicine and University Hospital Carl Gustav Carus, Technische Universität Dresden, Helmholtz-Zentrum Dresden-Rossendorf, 01307, Dresden, Germany
4 Department of Radiotherapy and Radiation Oncology, Faculty of Medicine and University Hospital Carl Gustav Carus, Technische Universität Dresden, 01307, Dresden, Germany
5 Helmholtz-Zentrum Dresden-Rossendorf, Institute of Radiooncology—OncoRay, 01328, Dresden, Germany
6 Department of Nuclear Medicine, Faculty of Medicine and University Hospital Carl Gustav Carus, Technische Universität Dresden, 01307, Dresden, Germany
7 Helmholtz-Zentrum Dresden-Rossendorf, Institute of Radiopharmaceutical Cancer Research, 01328, Dresden, Germany
8 School of Medicine, Koç University, 34450, Istanbul, Turkey
9 B CUBE—Center for Molecular Bioengineering, Technische Universität Dresden, 01307, Dresden, Germany
10 Department of Particle Therapy, University Hospital Essen, University of Duisburg-Essen, 45147, Essen, Germany
11 National Center for Tumor Diseases (NCT), Partner Site Dresden, Germany; German Cancer Research Center (DKFZ), Heidelberg, Germany; Faculty of Medicine and University Hospital Carl Gustav Carus, Technische Universität Dresden, Dresden, Germany, and; Helmholtz Association / Helmholtz-Zentrum Dresden - Rossendorf (HZDR), Dresden, Germany; 69192, Heidelberg, Germany

Supplementary method

Evaluation of cleaved caspase-3 and p21$^{cip1/waf1}$ positive cells on whole tumor sections

Cell detection

Images of whole section scanning of tumors, which immunofluorescence stained for cleaved caspase-3 and p21$^{cip1/waf1}$, were imported into QuPath. Whole tumor sections were annotated and cells within the annotations were automatically detected using the built-in function – positive cell detection. The parameters for cell detection were set as follows: background radius: 8 µm, median filter radius: 0 µm, sigma: 1.5 µm, minimum area: 20 µm^2, maximum area: 400 µm^2, DAPI intensity threshold: 100, cell expansion 10 µm. All cells and the corresponding mean fluorescence intensity measured in each compartment i.e. nucleus, cytoplasm, cell, were acquired.

Definition of thresholds for low, moderate and high fluorescence intensity.

Mean background fluorescence intensity of the markers in each image was calculated from mean gray value from five regions of interest (100 x 100 pixels), which were randomly selected from areas with negative signal of the markers. For each image, mean fluorescence intensity of the markers in each cell (cleaved caspase-3) or nucleus (p21$^{cip1/waf1}$) was subtracted with the corresponding mean background fluorescence intensity. Corrected mean fluorescence intensity was normalized by the min-max scaling method without considering negative corrected mean fluorescence intensity values. Ratio of cumulative sum and total sum of the normalized values was calculated for each cell. For cells or nucleus with negative corrected mean fluorescence intensity values, the ratio was defined as 0. Corrected mean fluorescence intensity values at the 10th (x_1), 37th(x_2), 63th (x_3) and 90th (x_4) percentile were identified in each image. Mean of corrected mean fluorescence intensity values at each percentile of the entire set of images were calculated. These corrected fluorescence intensity values were used as the fluorescence intensity thresholds for negative, low, intermediate, high, and overexposed.

Given y_i is mean fluorescence intensity of the markers in cells or nucleus (i):

$$y_i = \begin{cases} \text{Negative,} & y_i \le \bar{x}_1 \\ \text{Low,} & \bar{x}_1 > y_i \le \bar{x}_2 \\ \text{Intermediate,} & \bar{x}_2 > y_i \le \bar{x}_3 \\ \text{High,} & \bar{x}_3 > y_i \le \bar{x}_4 \\ \text{Overexposed,} & \text{otherwise} \end{cases}$$

Cells or nucleus with corrected mean fluorescence intensity of the markers that lower than or equal to $\overline{x_1}$ or greater than $\overline{x_4}$ were considered as negative and overexposed, respectively, to avoid the detection of false positive signal or staining artefacts.

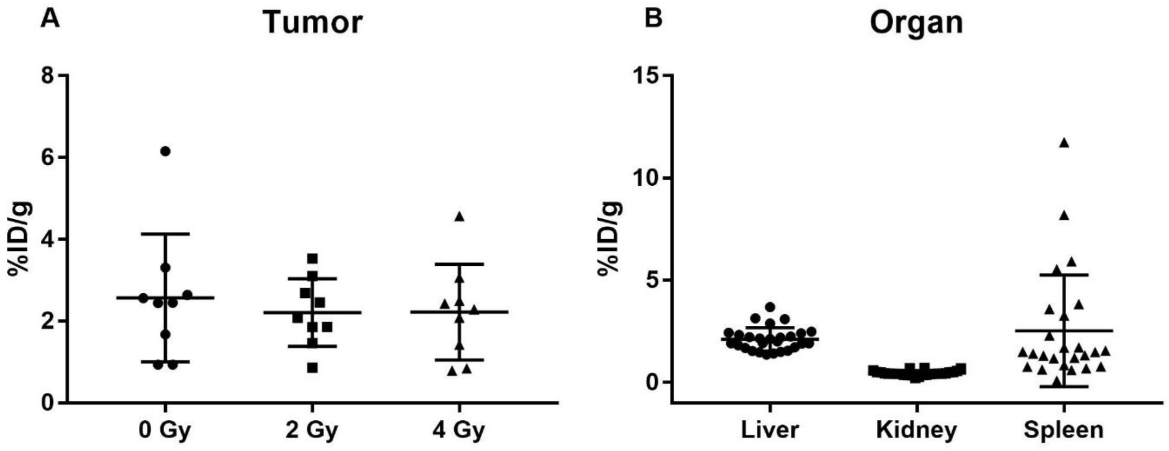

Figure S1 *In vivo* biodistribution of ^{90}Y-Cetuximab

In vivo biodistribution of ^{90}Y-Cetuximab administered intravenously 3 days prior to an external tumor irradiation of 0, 2 or 4 Gy. Tumors (N = 9/ treatment arm) and organs (liver: N = 27; kidney: N = 26; spleen: N = 25) were collected 24 h post irradiation. Strip charts show the percentage of uptake dose of injected dose per gram of the corresponding tissues (%ID/g) in tumors (A) and organs (B). One-way ANOVA followed by post-hoc test with Sidak's correction was applied to the uptake data in tumors. No statistically significant difference of radionuclide uptake was observed in tumors of the different arms. Horizontal lines and error bars represent mean and standard deviation.

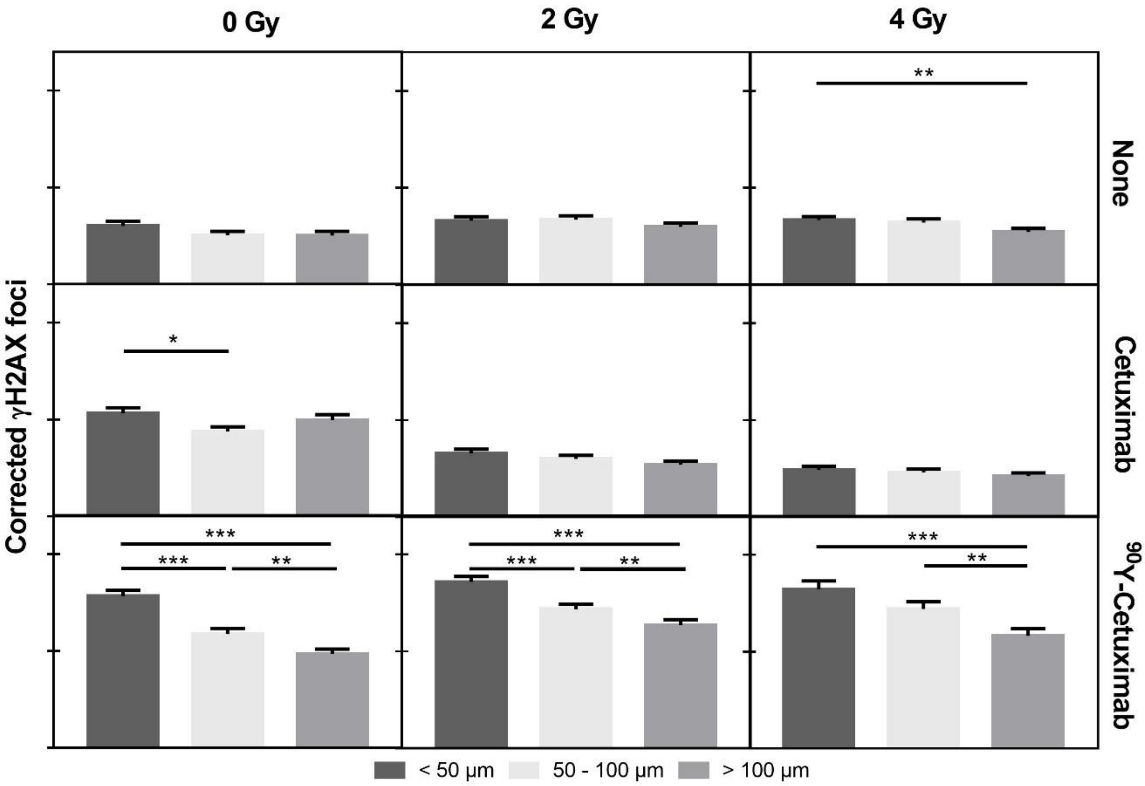

Figure S2 Corrected residual γH2AX foci plotted dependent on the distance from the nearest perfused vessel

Corrected residual γH2AX foci (cfoci) of $FaDu_{DD}$ xenograft tumors treated with monotherapy of external beam X-ray irradiation (0, 2, 4 Gy), Cetuximab or ^{90}Y-Cetuximab or the combination therapy. Data was plotted dependent on the distance to the nearest perfused vessel (< 50 µm, 50-100 µm, and > 100µm). Cells located in each distance group were randomly selected for manual foci counting. A linear mixed-effects model was performed to investigate the difference in cfoci among the distance within a treatment arm (*: $p < 0.05$, **: $p < 0.01$, ***: $p < 0.001$).

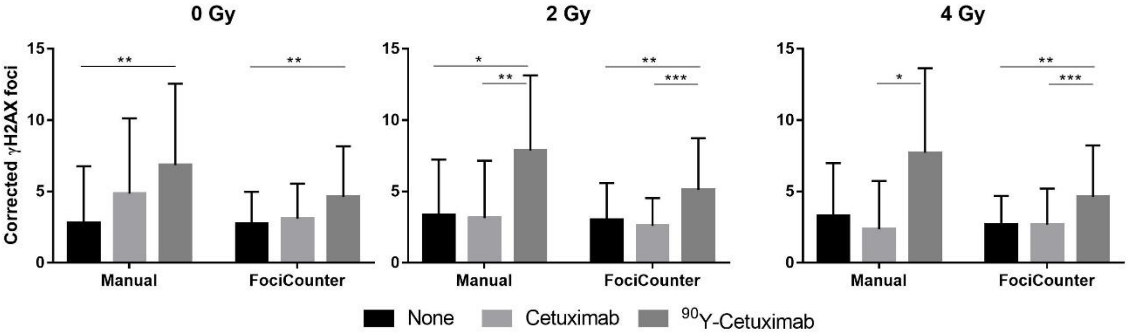

Figure S3 Corrected residual γH2AX foci determined by manual or FociCounter in nuclei located within the distance up to 100 μm from the nearest perfused vessel.

Residual γH2AX foci (cfoci) of $FaDu_{DD}$ xenograft tumors treated with monotherapy of external beam X-ray irradiation (0, 2, 4 Gy), Cetuximab or ^{90}Y-Cetuximab or combination therapy were counted manually or using FociCounter algorithm in nuclei located within the distance up to 100 μm from the nearest perfused vessel. A linear mixed-effects model with Sidak's correction for multiple comparison was performed (*: $p < 0.05$, **: $p < 0.01$, ***: $p < 0.001$).

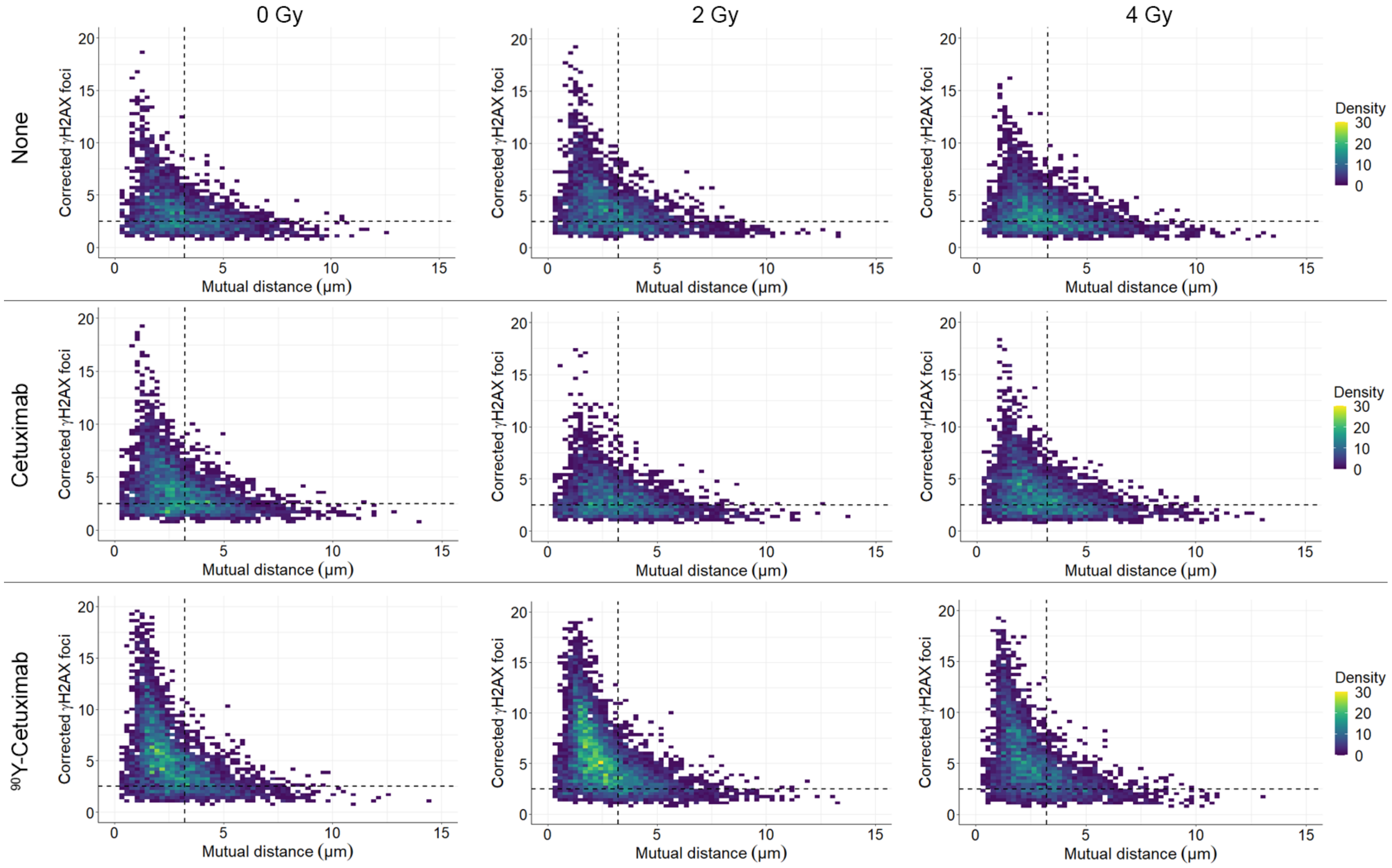

Figure S4. Two-dimensional density plot of corrected γH2AX foci number and mutual distance among foci determined by FociCounter, a FIJI ImageJ based algorithm. Tumor cells within the range of approx. 100 μm from the perfused vessels were automatically counted for foci number and distance among foci. Horizontal- and vertical dashed lines represent the mean of mutual distance among foci and corrected γH2AX foci of the untreated control group, respectively. The plots were done using RStudio with ggplot2 package.

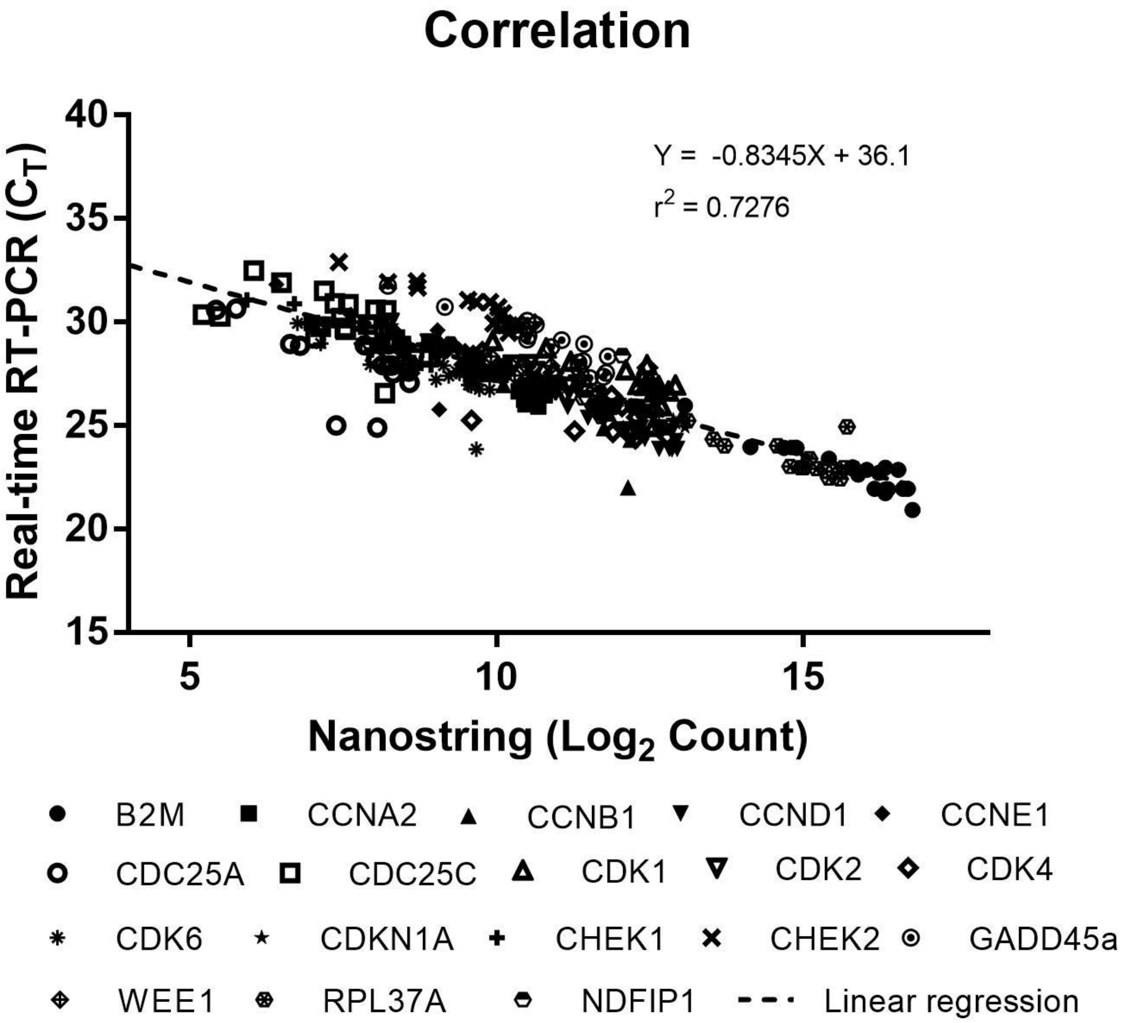

Figure S5 Correlation analysis of data from nanoString™ and real-time qRT-PCR

Negative proportional relationship between C_T values and log_2 Counts of 18 target genes assessed by real-time qRT-PCR and nanoString™, respectively. RNA from snap-frozen samples were isolated and analyzed for RNA expression using real-time qRT-PCR and nanoString™ (N = 18).

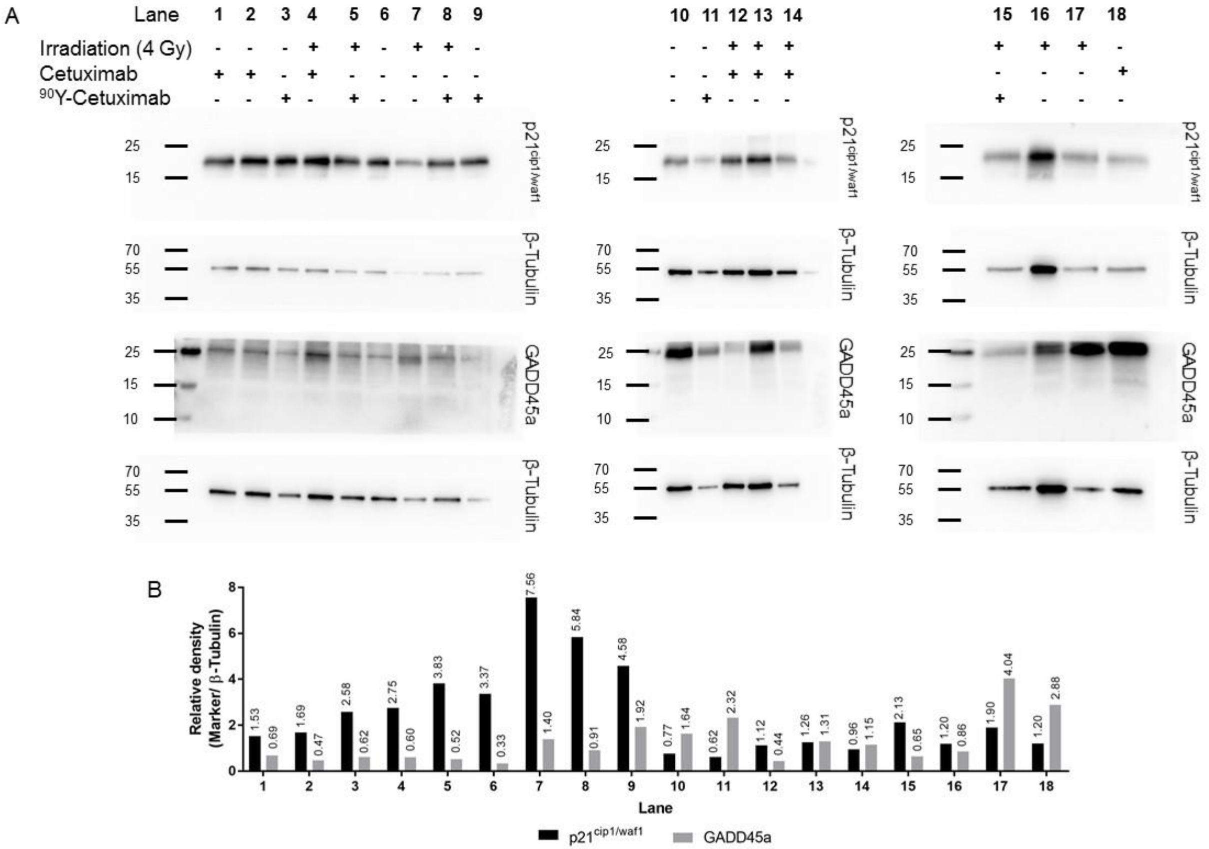

Figure S6 Full-length of immunoblotting images probed for GADD45α, p21$^{cip1/waf1}$, and β-Tubulin (A) and densitometry of each lane (B)

Proteins were isolated from FFPE tissue sections of FaDu$_{DD}$ tumors treated with monotherapy (external radiotherapy (0, 4 Gy), Cetuximab or ^{90}Y-Cetuximab) or the combination therapy. Total protein lysates were probed for GADD45α, p21$^{cip/waf1}$. β-Tubulin served as a reference protein for the determination of relative protein expression. The immunoblotting was performed under blinded condition (N=3). Immunoblotting was performed in six batches. Relative density of the probed proteins was calculated by normalizing the intensity of the markers with β-Tubulin, which was probed as reference for each batch of immunoblotting. Outlier test with robust regression and outlier removal method (Q=1%) was performed, by which the values from lane 7 (p21$^{cip1/waf1}$) and lane 17 (GADD45α) were excluded from the plot in figure 2D.

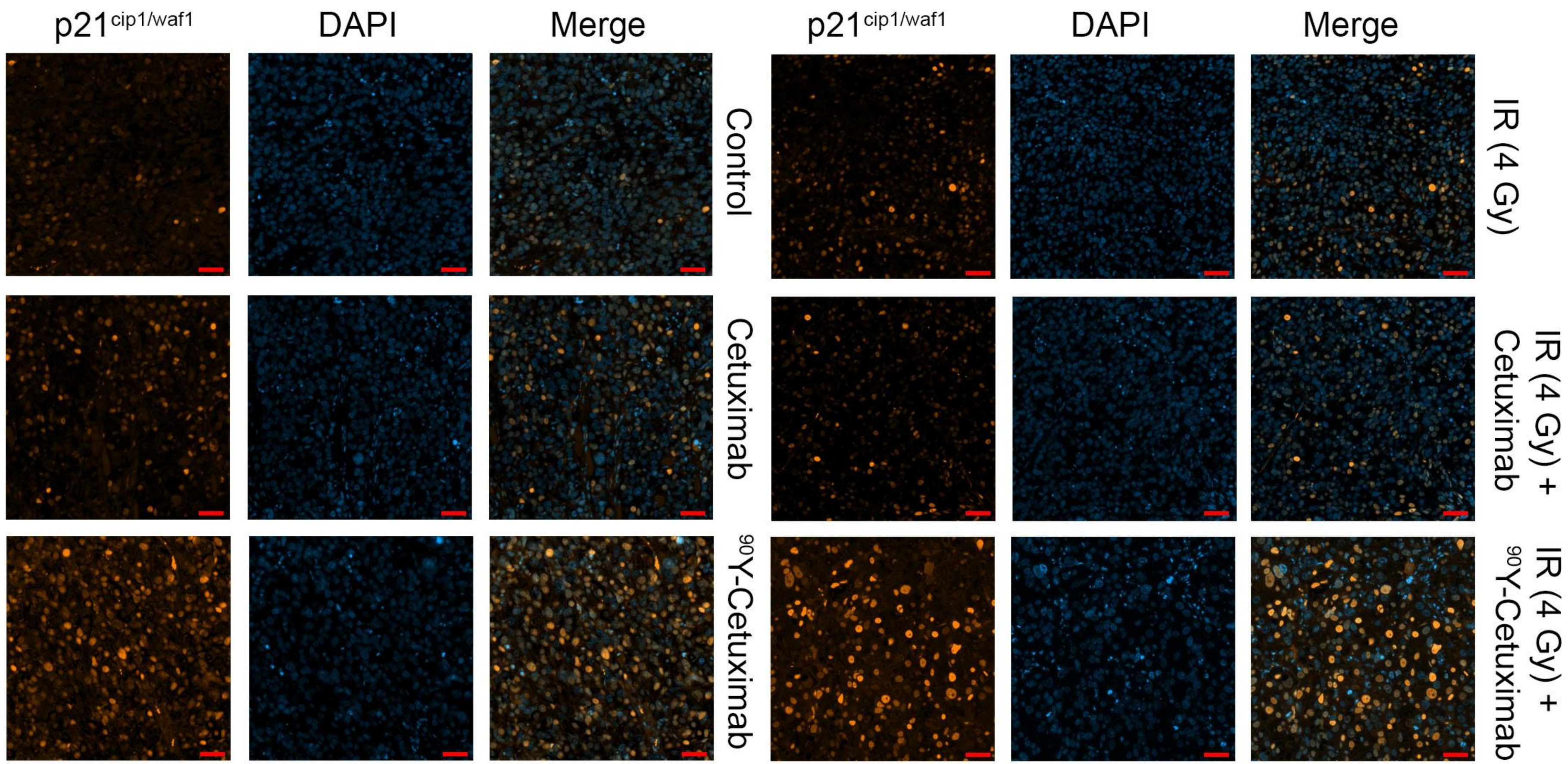

Figure S7 Representative images of immunofluorescence staining of p21$^{cip1/waf1}$

FaDu$_{DD}$ xenograft tumors were exposed to monotherapy (external radiotherapy, Cetuximab, or ^{90}Y-Cetuximab) or the combination therapy and formalin-fixed and paraffin-embedded 24 h post irradiation. FFPE tumor tissues were sectioned (3 µm) and immunofluorescence stained for p21$^{cip1/waf1}$ (orange) and counterstained with DAPI (blue). Scale bars (red): 50 µm.

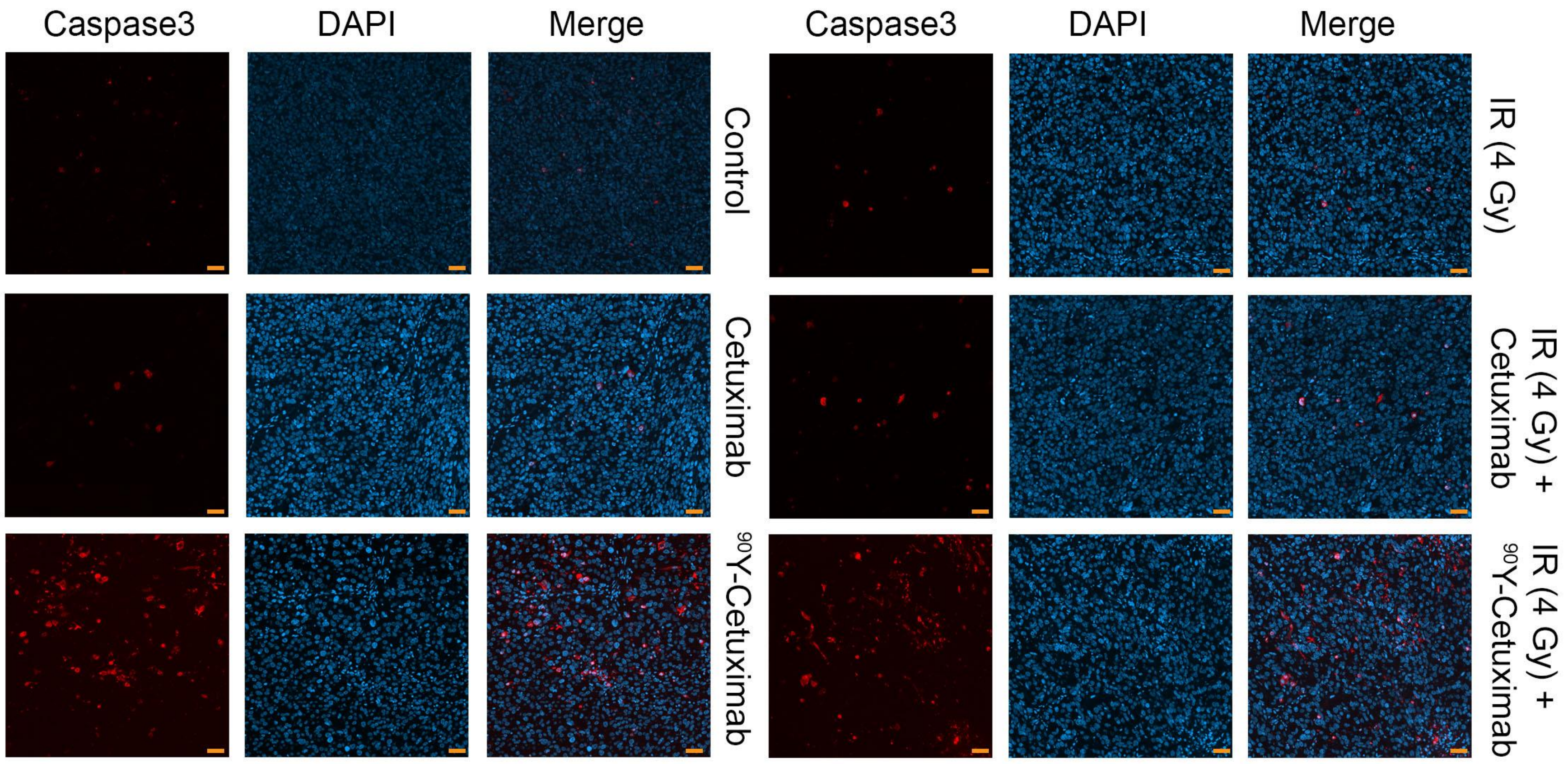

Figure S8 Representative images of immunofluorescence staining of cleaved-caspase 3

$FaDu_{DD}$ xenograft tumors exposed to monotherapy (external radiotherapy, Cetuximab, or ^{90}Y-Cetuximab) or the combination therapy were formalin-fixed and paraffin-embedded 24 h post irradiation. FFPE tumor tissues were sectioned (3 µm) and immunofluorescence stained for cleaved caspase-3 (red) and counterstained with DAPI (blue). Scale bars (orange): 50 µm.

Table S1 Total number of animals eligible for the study

Sample size [N]	X-Ray radiation dose		
	0 Gy	2 Gy	4 Gy
None	8	7	8
Cetuximab	9	8	8
^{90}Y-Cetuximab	9	9	9
	Total number of animals		75

Table S2 List of antibodies, kits, and chemicals used in this study

Antibodies

Description	Host	Catalog No.	Supplier	Dilution
Anti-Pimonidazole	Mouse	HP1-1000Kit	Natural Pharmacia International	1:50
Anti-Bromodeoxyuridine (clone: Bu20a)	Mouse	M0744	Agilent Dako	1:50
Anti-Cleaved Caspase-3 (Asp175)	Rabbit	9661S	Cell Signaling	1:200
Anti-GADD45α	Rabbit	ABE2696	Sigma-Aldrich	IF: 1:200<br>IB: 1:500
Anti-p21 Waf1/Cip1 (12D1)	Rabbit	2947S	Cell Signaling	IF: 1:200<br>IB: 1:1000
Anti-β-tubulin (9F3)	Rabbit	2128S	Cell Signaling	1:500
Anti-phospho-Histone H2A.X (Ser139), clone JBW301	Mouse	05-636-I	Sigma-Aldrich	1:1000
Anti-Rabbit IgG, Alexa Fluor 594	Goat	A-11012	Invitrogen	1:500
Anti-Rabbit IgG, DyLight 650	Donkey	SA5-10041	Invitrogen	1:200

IF: Immunofluorescence, IB: Immunoblotting

Kits

Description	Catalog No.	Supplier
ARK (Animal Research Kit) Peroxidase, Kit system	K395411-8	Agilent Dako
VECTASTAIN® Elite ABC-HRP Kit, Peroxidase (Rabbit IgG)	PK-6101	Vector Laboratories
TSA™ Kit #2 with HRP–goat anti-mouse IgG and Alexa Fluor® 488 Tyramide*	T20912	Invitrogen
RNeasy micro kit	#74004	Qiagen
Qubit™ RNA HS Assay Kit	#Q32852	Invitrogen

Chemicals

Description	Catalog No.	Supplier
Hematoxylin acid according to MAYER	10231	SAV Liquid Production
Eosin 1% in 96% Ethanol	-	University Hospital Dresden
4′,6-diamidino-2-phenylindole (1:1000)	D1306	Invitrogen
Cetuximab [Erbitux®]		University Hospital Dresden
Ketamin 500 (Curamed®)		CuraMed Pharma
Xylazine (Rompun®)		Bayer HealthCare
Bromodeoxyuridine	15240.02	SERVA electrophoresis
Pimonidazole	HP1-1000Kit	Natural Pharmacia International
TaqMan® Master Mix	#4444556	Applied Biosystems
Nuclease-Free water	#129114	Qiagen

Table S3 Customized gene set and the corresponding accession number for DNA damage response related mRNA expression analysis with nanoString™

Gene symbol	Accession number	Gene symbol	Accession number	Gene symbol	Accession number
ABL1	NM_005157.3	*FANCD2*	NM_033084.3	*PIK3R1*	NM_181504.2
AKT3	NM_005465.4	*FANCF*	NM_022725.2	*POLB*	NM_002690.1
APC	NM_000038.3	*FANCG*	NM_004629.1	*POLD1*	NM_002691.2
APEX1	NM_001641.2	*FANCI*	NM_001113378.1	*POLD3*	NM_006591.2
APEX2	NM_014481.2	*FANCL*	NM_001114636.1	*POLD4*	NM_021173.2
ATM	NM_138292.3	*FANCM*	NM_020937.2	*POLE2*	NM_002692.2
ATR	NM_001184.3	*FEN1*	NM_004111.4	*POLE4*	NM_019896.2
ATRIP	NM_130384.1	*GEN1*	NM_182625.3	*POLI*	NM_007195.2
AURKA	NM_003600.2	*GTF2H3*	NM_001516.3	*POLL*	NM_001174085.1
BCL2	NM_000657.2	*H2AFX*	NM_002105.2	*POLM*	NM_013284.3
BCL2L1	NM_138578.1	*HDAC1*	NM_004964.2	*POLQ*	NM_199420.3
BLM	NM_000057.2	*HDAC2*	NM_001527.1	*POLR2D*	NM_004805.3
BRCA1	NM_007305.2	*HLTF*	NM_139048.2	*POLR2H*	NM_001278698.1
BRCA2	NM_000059.3	*HUS1*	NM_004507.2	*POLR2J*	NM_006234.4
BRIP1	NM_032043.1	*KRAS*	NM_004985.3	*PRKACB*	NM_182948.2
BUB1B	NM_001211.4	*LIG1*	NM_000234.1	*PRKDC*	NM_006904.6
CASP8	NM_001228.4	*LIG3*	NM_002311.3	*PRKX*	NM_005044.1
CCND1	NM_053056.2	*LIG4*	NM_002312.3	*PTEN*	NM_000314.4
CCND2	NM_001759.2	*MAD2L2*	NM_001127325.1	*RAD1*	NM_133377.2
CCND3	NM_001760.2	*MDC1*	NM_014641.2	*RAD17*	NM_133338.1
CCNO	NM_021147.3	*MGMT*	NM_002412.3	*RAD18*	NM_020165.2
CDK7	NM_001799.2	*MNAT1*	NM_002431.2	*RAD21*	NM_006265.2
CDKN1A	NM_000389.2	*MPG*	NM_001015052.1	*RAD23A*	NM_005053.2
CDKN1B	NM_004064.2	*MRE11A*	NM_005591.3	*RAD23B*	NM_002874.3
CDKN2A	NM_000077.3	*MUTYH*	NM_012222.2	*RAD50*	NM_005732.2
CDKN2C	NM_001262.2	*MYC*	NM_002467.3	*RAD51*	NM_133487.2
CHEK1	NM_001114121.2	*MYD88*	NM_002468.3	*RAD51B*	NM_002877.5
CHEK2	NM_001005735.1	*NBN*	NM_001024688.1	*RAD51C*	NM_002876.2
CREBBP	NM_001079846.1	*NEIL1*	NM_024608.2	*RAD51D*	NM_002878.3
DDB1	NM_001923.3	*NEIL2*	NM_145043.2	*RAD52*	NM_134424.2
DDB2	NM_000107.1	*NEIL3*	NM_018248.2	*RAD54L*	NM_003579.2
EGFR	NM_201282.1	*NFKB1*	NM_003998.2	*RAD9A*	NM_004584.2
ERCC1	NM_001983.3	*NKX3-1*	NR_046072.1	*RB1*	NM_000321.1
ERCC2	NM_000400.2	*NLRP2*	NM_017852.1	*RECQL*	NM_032941.2
ERCC3	NM_000122.1	*NTHL1*	NM_002528.5	*RECQL5*	NM_004259.6
ERCC4	NM_005236.2	*OGG1*	NM_002542.5	*REV1*	NM_016316.2
ERCC5	NM_000123.2	*PARP1*	NM_001618.3	*RFC1*	NM_001204747.1
ERCC6	NM_000124.2	*PARP2*	NM_005484.3	*RFC3*	NM_002915.3
ERCC8	NM_000082.3	*PARP3*	NM_005485.4	*RFC4*	NM_181573.2
FAN1	NM_001146094.1	*PARP4*	NM_006437.3	*RMI1*	NM_024945.2
FANCA	NM_000135.2	*PCNA*	NM_002592.2	*RMI2*	NM_152308.1
FANCB	NM_152633.2	*PIK3CA*	NM_006218.2	*RPA1*	NM_002945.3
FANCC	NM_000136.2	*PIK3CB*	NM_006219.1	*RPA3*	NM_002947.3

Gene symbol	Accession number	Gene symbol	Accession number	Gene symbol	Accession number
RPS27A	NM_002954.5	*MCM2*	NM_004526.4	*NDFIP1*	NM_030571.3
RRM2B	NM_015713.3	*RBL2*	NM_001323608.2	*POLR2A*	NM_000937.4
SIRT1	NM_012238.4	*CDK5R1*	NM_003885.3	*RPL11*	NM_000975.3
SLFN11	NM_001104587.1	*BIRC5*	NM_001168.3	*RPL37A*	NM_000998.4
SLK	NM_014720.2	*CDC34*	NM_004359.2		
SLX4	NM_032444.2	*MCM5*	NM_006739.4		
SMAD4	NM_005359.3	*CDC25A*	NM_001789.2		
SMARCA4	NM_003072.3	*CDC20*	NM_001255.3		
SMC1A	NM_006306.2	*RBBP8*	NM_002894.3		
SMC3	NM_005445.3	*MAD2L1*	NM_002358.4		
SMUG1	NM_001243789.1	*CCNF*	NM_001761.3		
SUMO3	NM_006936.2	*ANAPC2*	NM_013366.4		
TDG	NM_003211.4	*CDKN2B*	NM_004936.4		
TIPIN	NM_017858.2	*CCNG1*	NM_199246.2		
TOP3A	NM_004618.3	*TFDP1*	NM_007111.5		
TOP3B	NM_003935.4	*GADD45A*	NM_001924.4		
TP53	NM_000546.2	*MKI67*	NM_002417.4		
TP53BP1	NM_001141980.1	*CUL2*	NM_001198778.2		
TREX1	NM_016381.3	*CKS2*	NM_001827.3		
UBB	NM_018955.2	*CDKN3*	NM_005192.3		
UBE2T	NM_014176.3	*CCNA2*	NM_001237.3		
UNG	NM_003362.3	*CDK4*	NM_000075.3		
USP1	NM_003368.4	*E2F1*	NM_005225.1		
WEE1	NM_003390.3	*E2F4*	NM_001950.4		
WRN	NM_000553.4	*CDK6*	NM_001259.6		
XPA	NM_000380.3	*CASP3*	NM_001284409.1		
XPC	NM_004628.3	*CCNH*	NM_001199189.1		
XRCC1	NM_006297.2	*CUL1*	NM_001370661.1		
XRCC2	NM_005431.1	*KPNA2*	NM_001320611.1		
XRCC3	NM_001100119.1	*MCM3*	NM_001270472.3		
XRCC4	NM_003401.3	*SKP2*	NM_005983.4		
XRCC5	NM_021141.3	*TFDP2*	NM_001178138.2		
XRCC6	NM_001469.3	*CDC25C*	NM_001790.4		
CDK2	NM_001798.4	*KNTC1*	NM_014708.6		
CKS1B	NM_001826.3	*CCNB2*	NM_004701.4		
CUL3	NM_001257198.2	*STMN1*	NM_001145454.3		
MDM2	NM_001145337.2	*CCNG2*	NM_004354.3		
CCNB1	NM_031966.3	*RBL1*	NM_002895.5		
GTSE1	NM_016426.7	*CCNT1*	NM_001240.4		
CDC16	NM_001078645.3	*CCNE1*	NM_001238.3		
CDK5RAP1	NM_016408.4	*CDK1*	NM_001786.4		
MCM4	NM_005914.4	*BCCIP*	NM_016567.4		
CDK8	NM_001260.3	*SERTAD1*	NM_013376.4		
CCNC	NM_005190.4	*ACTR3*	NM_001277140.1		
CDC6	NM_001254.4	*B2M*	NM_004048.2		
AURKB	NM_001313950.2	*GNB2L1*	NM_006098.4		

Table S4 Primer list for real-time qRT-PCR

Gene Symbol	Gene Name	UniGene ID	Amplicon Length	Dye	Assay ID
B2M	Beta-2-microglobulin	Hs.534255	64	FAM-MGB	Hs00187842_m1
NDFIP1	Nedd4 family interacting protein 1	Hs.653510	67	FAM-MGB	Hs00228968_m1
RPL37A	Ribosomal protein L37a	Hs.433701	125	FAM-MGB	Hs01102345_m1
CDK6	Cyclin dependent kinase 6	Hs.119882	64	FAM-MGB	Hs01026371_m1
CDK4	Cyclin dependent kinase 4	Hs.95577	75	FAM-MGB	Hs00364847_m1
CDK1	Cyclin dependent kinase 1	Hs.732435	109	FAM-MGB	Hs00938777_m1
CDK2	Cyclin dependent kinase 2	Hs.19192	58	FAM-MGB	Hs01548894_m1
CCNB1	Cyclin B1	Hs.23960	86	FAM-MGB	Hs01030099_m1
CCND1	Cyclin D1	Hs.523852	57	FAM-MGB	Hs00765553_m1
CCNE1	Cyclin E1	Hs.244723	64	FAM-MGB	Hs01026536_m1
CCNA2	Cyclin A2	Hs.58974	81	FAM-MGB	Hs00996788_m1
CDC25A	Cell division cycle 25A	Hs.437705	68	FAM-MGB	Hs00947994_m1
CDC25C	Cell division cycle 25C	Hs.656	145	FAM-MGB	Hs00156411_m1
WEE1	WEE1 G2 checkpoint kinase	Hs.249441	70	FAM-MGB	Hs01119384_g1
CHEK2	Checkpoint kinase 2	Hs.291363	109	FAM-MGB	Hs00200485_m1
CHEK1	Checkpoint kinase 1	Hs.24529	79	FAM-MGB	Hs00967506_m1
GADD45A	Growth arrest and DNA damage inducible alpha	Hs.80409	123	FAM-MGB	Hs00169255_m1
CDKN1A	Cyclin dependent kinase inhibitor 1A	Hs.370771	66	FAM-MGB	Hs00355782_m1

All pre-designed primers were purchased from Invitrogen (Darmstadt, Germany)

Table S5 Summary of descriptive statistics from manual γH2AX foci determination

Radiation dose [Gy]	Cetuximab	Range from perfused vessel [μm]	Number of analyzed cells	Nucleus area [μm2]					Corrected γH2AX foci				
				Mean	Std. Error	Std. Deviation	Min	Max	Mean	Std. Error	Std. Deviation	Min	Max
0 Gy	None	< 50	335	116.85	1.76	32.00	37.35	263.86	3.02	0.24	4.32	0.00	21.94
		50 - 100	335	112.26	1.66	30.13	34.74	235.48	2.54	0.20	3.62	0.00	17.83
		> 100	335	111.51	1.73	31.36	28.28	256.78	2.25	0.22	3.93	0.00	21.68
	unlabeled	< 50	390	118.77	1.68	33.18	59.17	263.67	5.33	0.29	5.68	0.00	28.00
		50 - 100	390	115.24	1.66	32.87	56.68	305.21	4.38	0.24	4.79	0.00	24.39
		> 100	385	114.35	1.58	31.02	54.99	255.97	4.98	0.28	5.48	0.00	26.85
	^{90}Y-labeled	< 50	380	147.82	3.00	58.56	41.07	422.71	7.84	0.30	5.79	0.00	32.52
		50 - 100	380	132.53	2.53	49.36	48.48	420.00	5.88	0.28	5.44	0.00	31.32
		> 100	380	125.88	2.25	43.93	57.88	412.71	4.85	0.24	4.71	0.00	23.23
2 Gy	None	< 50	350	119.07	1.92	35.94	49.66	352.79	3.30	0.22	4.07	0.00	20.04
		50 - 100	350	115.91	1.68	31.52	54.30	250.24	3.36	0.20	3.74	0.00	20.39
		> 100	350	117.3	1.75	32.76	59.02	293.22	3.00	0.20	3.66	0.00	17.22
	unlabeled	< 50	405	124.11	1.76	35.37	58.70	269.77	3.29	0.22	4.33	0.00	22.62
		50 - 100	405	119.51	1.93	38.80	44.09	309.46	3.00	0.18	3.66	0.00	17.85
		> 100	405	116.10	1.83	36.90	46.32	328.79	2.71	0.18	3.62	0.00	21.93
	^{90}Y-labeled	< 50	370	148.05	2.82	54.19	33.36	403.01	8.59	0.28	5.40	0.00	26.32
		50 - 100	370	141.23	2.98	57.37	44.81	527.86	7.18	0.26	5.02	0.00	29.19
		> 100	370	133.38	2.90	55.81	53.46	477.83	6.36	0.28	5.31	0.00	30.40
4 Gy	None	< 50	385	118.90	1.69	33.13	52.85	286.55	3.34	0.18	3.72	0.00	22.72
		50 - 100	385	115.02	1.80	35.31	44.96	316.27	3.21	0.19	3.71	0.00	23.11
		> 100	385	112.08	1.68	32.98	44.89	246.87	2.73	0.19	3.63	0.00	23.31
	unlabeled	< 50	345	120.99	1.69	31.36	55.05	258.10	2.44	0.19	3.52	0.00	28.54
		50 - 100	345	117.17	1.83	34.04	56.23	314.66	2.31	0.17	3.22	0.00	25.55
		> 100	345	115.95	1.88	34.97	52.72	281.52	2.11	0.17	3.16	0.00	19.43
	^{90}Y-labeled	< 50	215	145.19	3.86	56.66	49.60	473.35	8.23	0.43	6.28	0.00	40.30
		50 - 100	215	135.42	3.54	51.93	61.25	330.88	7.20	0.38	5.53	0.00	27.38
		> 100	215	129.53	3.02	44.24	56.31	326.14	5.82	0.37	5.39	0.00	33.90

Table S6 Statistical output of corrected foci and nucleus area analyzed by a linear mixed-effects model based on the manual assessment of γH2AX foci

	Radiation dose [Gy]	Range [μm]	Cetuximab (I)	Cetuximab (J)					
				Unlabeled			^{90}Y-labeled		
				Mean difference (I-J)	Std. Error	p value	Mean difference (I-J)	Std. Error	p value
Corrected foci[a]	0 Gy	< 50	none	-0.59	0.38	0.339	-1.33	0.38	**0.003**
			unlabeled	-	-	-	-0.74	0.37	0.140
		50 - 100	none	-0.54	0.36	0.363	-0.99	0.36	**0.022**
			unlabeled	-	-	-	-0.45	0.35	0.481
		> 100	none	-0.70	0.35	0.147	-0.80	0.35	0.078
			unlabeled	-	-	-	-0.10	0.34	0.987
	2 Gy	< 50	none	0.09	0.38	0.994	-1.37	0.38	**0.002**
			unlabeled	-	-	-	-1.46	0.37	**0.001**
		50 - 100	none	0.18	0.36	0.942	-1.03	0.36	**0.016**
			unlabeled	-	-	-	-1.21	0.35	**0.002**
		> 100	none	0.12	0.35	0.982	-0.93	0.35	**0.029**
			unlabeled	-	-	-	-1.05	0.34	**0.009**
	4 Gy	< 50	none	0.35	0.38	0.739	-1.13	0.42	**0.028**
			unlabeled	-	-	-	-1.49	0.44	**0.003**
		50 - 100	none	0.28	0.36	0.815	-1.04	0.40	**0.031**
			unlabeled	-	-	-	-1.33	0.41	**0.005**
		> 100	none	0.24	0.35	0.879	-0.88	0.39	0.078
			unlabeled	-	-	-	-1.11	0.40	**0.020**
Nucleus area[b]	0 Gy	< 50	none	-0.01	0.02	0.964	-0.09	0.02	**<0.001**
			unlabeled	-	-	-	-0.08	0.02	**<0.001**
		50 - 100	none	-0.01	0.02	0.917	-0.06	0.02	**0.005**
			unlabeled	-	-	-	-0.05	0.02	**0.021**
		> 100	none	-0.01	0.02	0.901	-0.05	0.02	0.086
			unlabeled	-	-	-	-0.04	0.02	0.274
	2 Gy	< 50	none	-0.02	0.02	0.713	-0.08	0.02	**<0.001**
			unlabeled	-	-	-	-0.07	0.02	**0.003**
		50 - 100	none	-0.01	0.02	0.969	-0.07	0.02	**0.002**
			unlabeled	-	-	-	-0.06	0.02	**0.005**
		> 100	none	0.01	0.02	0.975	-0.04	0.02	0.163
			unlabeled	-	-	-	-0.05	0.02	0.058
	4 Gy	< 50	none	-0.01	0.02	0.956	-0.07	0.02	**0.006**
			unlabeled	-	-	-	-0.06	0.02	**0.025**
		50 - 100	none	-0.01	0.02	0.917	-0.06	0.02	**0.031**
			unlabeled	-	-	-	-0.05	0.02	0.122
		> 100	none	-0.02	0.02	0.868	-0.06	0.02	0.078
			unlabeled	-	-	-	-0.04	0.03	0.301

Square root transformation of corrected foci [a] and log-transformation of nucleus area [b] were performed. The statistical analysis was done with a linear mixed-effects model followed by Sidak's correction for the multiple comparison. Mean difference (I-J) represents the difference of marginal means estimated from the linear mixed effects model.

Table S7 Top 20 differentially expressed DNA damage response related genes in the treated groups relative to the untreated group determined by nSolver™

	**Genes**	**Log2 fold change**	**Std error (log2)**	**Lower confidence limit (log2)**	**Upper confidence limit (log2)**	**p-value**	**Adj. p value**
**Unlabeled Cetuximab vs. Control**	*XPC*	0.82	0.22	0.39	1.25	0.00108	0.527
	CCND1	-0.43	0.12	-0.66	-0.20	0.00114	0.527
	XRCC1	-0.34	0.09	-0.53	-0.16	0.00122	0.527
	RBL2	0.38	0.12	0.16	0.61	0.00304	0.833
	CHEK2	-0.32	0.10	-0.52	-0.13	0.00322	0.833
	MCM3	-0.47	0.15	-0.77	-0.17	0.0048	1
	CDK8	-0.26	0.08	-0.42	-0.09	0.00564	1
	NTHL1	-0.41	0.14	-0.67	-0.14	0.00669	1
	CCNO	-1.40	0.49	-2.34	-0.43	0.00874	1
	CDK5R1	-0.47	0.16	-0.79	-0.14	0.00912	1
	CKS2	-0.33	0.12	-0.57	-0.09	0.0116	1
	CCNF	-0.55	0.20	-0.95	-0.15	0.0121	1
	LIG4	0.31	0.12	0.08	0.53	0.0136	1
	PARP4	0.27	0.10	0.07	0.47	0.0137	1
	CDK1	-0.42	0.16	-0.73	-0.11	0.015	1
	RB1	-0.20	0.08	-0.34	-0.05	0.0157	1
	MCM5	-0.51	0.20	-0.89	-0.12	0.0166	1
	CDK2	-0.33	0.13	-0.58	-0.08	0.0175	1
	CCNB1	-0.60	0.24	-1.07	-0.13	0.0191	1
	AURKB	-0.54	0.22	-0.97	-0.11	0.0213	1
**^{90}Y-Cetuximab vs. Control**	*GADD45A*	1.18	0.14	0.90	1.45	1.35E-08	**1.74E-05**
	CDKN1A	1.22	0.16	0.90	1.53	7.96E-08	**5.15E-05**
	PTEN	-0.63	0.11	-0.86	-0.41	9.55E-06	**0.00412**
	CDKN2C	-1.05	0.21	-1.47	-0.63	4.91E-05	**0.0136**
	SKP2	-0.59	0.12	-0.82	-0.35	5.27E-05	**0.0136**
	NKX3-1	0.89	0.20	0.51	1.29	0.000145	**0.0249**
	LIG4	0.51	0.12	0.29	0.74	0.000158	**0.0249**
	CCND1	0.52	0.12	0.29	0.74	0.000159	**0.0249**
	MDM2	0.53	0.12	0.29	0.76	0.000198	**0.0249**
	CCNE1	0.66	0.15	0.36	0.96	0.000207	**0.0249**
	CCND3	0.48	0.11	0.27	0.70	0.000211	**0.0249**
	PARP3	0.63	0.15	0.34	0.92	0.000261	**0.0281**
	RECQL	0.45	0.11	0.24	0.67	0.000393	**0.0375**
	SERTAD1	0.43	0.10	0.22	0.67	0.000406	**0.0375**
	POLD4	0.49	0.12	0.25	0.74	0.000601	0.0518
	CASP8	0.56	0.15	0.26	0.86	0.00115	0.0932
	MYD88	0.94	0.26	0.43	1.45	0.00132	0.0953
	RAD23A	-0.23	0.06	-0.35	-0.11	0.00133	0.0953
	PIK3R1	0.43	0.12	0.20	0.67	0.00141	0.0961
	ERCC4	0.61	0.18	0.27	0.96	0.002	0.129

Table S7. *continued.*

	Genes	Log2 fold change	Std error (log2)	Lower confidence limit (log2)	Upper confidence limit (log2)	p-value	Adj. p value
**Irradiation (4Gy) vs. Control**	*SKP2*	-0.51	0.12	-0.74	-0.27	0.000282	0.284
	CDKN1A	0.62	0.16	0.31	0.94	0.00073	0.284
	GADD45A	0.54	0.14	0.27	0.82	0.000748	0.284
	LIG4	0.44	0.12	0.21	0.66	0.000878	0.284
	MDM2	0.39	0.12	0.15	0.62	0.00379	0.981
	SERTAD1	0.31	0.10	0.10	0.51	0.0071	1
	PARP4	0.29	0.10	0.09	0.49	0.00811	1
	TREX1	-0.33	0.13	-0.58	-0.08	0.0159	1
	BCL2L1	0.47	0.18	0.11	0.83	0.0176	1
	PARP3	0.37	0.15	0.08	0.66	0.0203	1
	CCNF	-0.50	0.20	-0.90	-0.10	0.0211	1
	NFKB1	0.29	0.12	0.06	0.52	0.0217	1
	PTEN	-0.26	0.11	-0.48	-0.04	0.0297	1
	CCNE1	0.35	0.15	0.05	0.65	0.0304	1
	MKI67	-0.46	0.20	-0.86	-0.06	0.034	1
	TP53	0.78	0.35	0.09	1.46	0.037	1
	MYC	0.20	0.10	0.01	0.39	0.0469	1
	PIK3CA	0.28	0.14	0.01	0.54	0.0505	1
	PCNA	-0.26	0.13	-0.51	-0.01	0.0524	1
	POLD4	0.25	0.13	0.01	0.50	0.0525	1
**Irradiation (4Gy) + Unlabeled Cetuximab vs. Control**	*RBL2*	0.49	0.12	0.27	0.72	0.000288	0.175
	LIG4	0.48	0.12	0.26	0.70	0.000335	0.175
	CCNF	-0.84	0.20	-1.24	-0.44	0.000406	0.175
	MCM3	-0.53	0.15	-0.82	-0.23	0.00191	0.619
	MKI67	-0.67	0.20	-1.07	-0.27	0.0031	0.647
	SERTAD1	0.34	0.10	0.14	0.54	0.00329	0.647
	XPC	0.72	0.22	0.28	1.15	0.0035	0.647
	CKS2	-0.38	0.12	-0.61	-0.14	0.00443	0.716
	XRCC1	-0.29	0.09	-0.47	-0.10	0.00545	0.783
	SKP2	-0.36	0.12	-0.59	-0.13	0.00611	0.79
	PARP4	0.28	0.10	0.08	0.48	0.0101	1
	HDAC2	0.55	0.20	0.16	0.94	0.0106	1
	E2F4	-0.29	0.11	-0.50	-0.08	0.0115	1
	PARP3	0.40	0.15	0.11	0.69	0.0123	1
	CDKN1A	0.43	0.16	0.11	0.74	0.014	1
	CDK2	-0.34	0.13	-0.59	-0.08	0.0157	1
	MDM2	0.31	0.12	0.08	0.55	0.0157	1
	RMI1	-0.24	0.09	-0.43	-0.06	0.017	1
	SLFN11	-0.43	0.17	-0.75	-0.10	0.0185	1
	PIK3CA	0.34	0.13	0.07	0.60	0.0192	1

Table S7. *continued.*

	Genes	Log2 fold change	Std error (log2)	Lower confidence limit (log2)	Upper confidence limit (log2)	p-value	Adj. p value
**Irradiation (4Gy) + ^{90}Y-Cetuximab vs. Control**	*GADD45A*	1.23	0.14	0.96	1.51	5.95E-09	**7.70E-06**
	CDKN1A	1.09	0.16	0.78	1.41	4.74E-07	**0.000306**
	MDM2	0.80	0.12	0.56	1.03	7.40E-07	**0.000319**
	LIG4	0.74	0.11	0.52	0.96	9.96E-07	**0.000322**
	SKP2	-0.76	0.12	-0.99	-0.52	1.70E-06	**0.000439**
	PTEN	-0.64	0.11	-0.86	-0.42	8.55E-06	**0.00184**
	NTHL1	-0.76	0.14	-1.04	-0.49	1.28E-05	**0.00237**
	RAD23A	-0.33	0.06	-0.46	-0.21	2.02E-05	**0.00327**
	PARP3	0.72	0.15	0.43	1	5.54E-05	**0.00796**
	POLD4	0.60	0.12	0.35	0.84	6.96E-05	**0.009**
	UBE2T	-0.77	0.16	-1.09	-0.45	9.15E-05	**0.0108**
	CDKN2C	-0.96	0.21	-1.37	-0.54	0.000152	**0.0164**
	STMN1	-0.62	0.14	-0.90	-0.34	0.000218	**0.0216**
	RPA1	-0.44	0.10	-0.64	-0.24	0.000273	**0.0216**
	PCNA	-0.54	0.13	-0.79	-0.29	0.000276	**0.0216**
	AURKA	-0.85	0.20	-1.24	-0.46	0.000297	**0.0216**
	MKI67	-0.86	0.20	-1.26	-0.46	0.000301	**0.0216**
	SERTAD1	0.44	0.10	0.23	0.64	0.000301	**0.0216**
	SUMO3	-0.42	0.10	-0.62	-0.22	0.000343	**0.0234**
	PARP1	-0.80	0.19	-1.18	-0.42	0.000365	**0.0236**

Table S8 Functional annotation analysis of genes that significantly up- and downregulated upon monotherapy of ^{90}Y-Cetuximab

	Term	Count	%	p value	Genes	List Total	Pop Hits	Fold Enrichment	Adjusted p-values (Benjamini-Hochberg)	FDR
Upregulation	**hsa04115: p53 signaling pathway**	**6**	**54.55**	**4.12E-09**	***CCND3, CDKN1A, CCND1, CCNE1, GADD45A, MDM2***	**9**	**67**	**68.45**	**1.77E-07**	**1.24E-07**
	**hsa04110: Cell cycle**	**6**	**54.55**	**9.42E-08**	***CCND3, CDKN1A, CCND1, CCNE1, GADD45A, MDM2***	**9**	**124**	**36.98**	**2.02E-06**	**1.41E-06**
	**hsa05215: Prostate cancer**	**5**	**45.45**	**1.68E-06**	***CDKN1A, CCND1, CCNE1, MDM2, NKX3-1***	**9**	**88**	**43.43**	**2.41E-05**	**1.68E-05**
	**hsa05203: Viral carcinogenesis**	**5**	**45.45**	**4.88E-05**	***CCND3, CDKN1A, CCND1, CCNE1, MDM2***	**9**	**205**	**18.64**	**5.25E-04**	**3.66E-04**
	**hsa04151: PI3K-Akt signaling pathway**	**5**	**45.45**	**3.71E-04**	***CCND3, CDKN1A, CCND1, CCNE1, MDM2***	**9**	**345**	**11.08**	**0.0027**	**0.0019**
	**hsa04068: FoxO signaling pathway**	**4**	**36.36**	**3.77E-04**	***CDKN1A, CCND1, GADD45A, MDM2***	**9**	**134**	**22.82**	**0.0027**	**0.0019**
	**hsa05200: Pathways in cancer**	**5**	**45.45**	**6.11E-04**	***CDKN1A, CCND1, CCNE1, MDM2, NKX3-1***	**9**	**393**	**9.72**	**0.0037**	**0.0026**
	**hsa05219: Bladder cancer**	**3**	**27.27**	**9.49E-04**	***CDKN1A, CCND1, MDM2***	**9**	**41**	**55.93**	**0.0051**	**0.0036**
	**hsa05214: Glioma**	**3**	**27.27**	**0.0024**	***CDKN1A, CCND1, MDM2***	**9**	**65**	**35.28**	**0.0113**	**0.0079**
	**hsa05218: Melanoma**	**3**	**27.27**	**0.0028**	***CDKN1A, CCND1, MDM2***	**9**	**71**	**32.30**	**0.0113**	**0.0079**
	**hsa05220: Chronic myeloid leukemia**	**3**	**27.27**	**0.0029**	***CDKN1A, CCND1, MDM2***	**9**	**72**	**31.84**	**0.0113**	**0.0079**
	**hsa05206: MicroRNAs in cancer**	**4**	**36.36**	**0.0034**	***CDKN1A, CCND1, CCNE1, MDM2***	**9**	**286**	**10.69**	**0.0122**	**0.0085**
	**hsa05162: Measles**	**3**	**27.27**	**0.0096**	***CCND3, CCND1, CCNE1***	**9**	**133**	**17.24**	**0.0318**	**0.0222**
	**hsa05161: Hepatitis B**	**3**	**27.27**	**0.0114**	***CDKN1A, CCND1, CCNE1***	**9**	**145**	**15.81**	**0.0349**	**0.0243**
	**hsa05205: Proteoglycans in cancer**	**3**	**27.27**	**0.0210**	***CDKN1A, CCND1, MDM2***	**9**	**200**	**11.47**	**0.0601**	**0.0419**
	hsa05166: HTLV-I infection	3	27.27	0.0328	*CCND3, CDKN1A, CCND1*	9	254	9.028	0.0882	0.0615
	hsa05222: Small cell lung cancer	2	18.18	0.0947	*CCND1, CCNE1*	9	85	17.98	0.2396	0.1671
Downregulation	hsa05222: Small cell lung cancer	2	66.67	0.0246	*PTEN, SKP2*	3	85	53.95	0.3215	0.3215
	hsa04110: Cell cycle	2	66.67	0.0357	*CDKN2C, SKP2*	3	124	36.98	0.3215	0.3215
	hsa04068: FoxO signaling pathway	2	66.67	0.0386	*PTEN, SKP2*	3	134	34.22	0.3215	0.3215

* The analysis was performed using an online tool for functional annotation (DAVID Bioinformatics Resources 6.8). KEGG pathway was used as the reference database.

Table S9 Functional annotation analysis of genes that significantly up- and downregulated upon the combination therapy of ^{90}Y-Cetuximab and external tumor irradiation of 4 Gy

	Term	Count	%	p value	Genes	List Total	Pop Hits	Fold Enrichment	Adjusted p-values (Benjamini-Hochberg)	FDR
Upregulation	**hsa03420: Nucleotide excision repair**	**3**	**27.27**	**0.0012**	***POLD4, ERCC4, ERCC6***	**9**	**47**	**48.79**	**0.0416**	**0.0329**
	**hsa04115: p53 signaling pathway**	**3**	**27.27**	**0.0025**	***CDKN1A, GADD45A, MDM2***	**9**	**67**	**34.22**	**0.0416**	**0.0329**
	**hsa05220: Chronic myeloid leukemia**	**3**	**27.27**	**0.0029**	***CDKN1A, MDM2, BCL2L1***	**9**	**72**	**31.85**	**0.0416**	**0.0329**
	hsa04110: Cell cycle	3	27.27	0.0084	*CDKN1A, GADD45A, MDM2*	9	124	18.49	0.0840	0.0664
	hsa04068: FoxO signaling pathway	3	27.27	0.0098	*CDKN1A, GADD45A, MDM2*	9	134	17.11	0.0840	0.0664
	hsa05202: Transcriptional misregulation in cancer	3	27.27	0.0149	*CDKN1A, MDM2, BCL2L1*	9	167	13.73	0.1068	0.0844
	hsa05166: HTLV-I infection	3	27.27	0.0328	*POLD4, CDKN1A, BCL2L1*	9	254	9.03	0.2016	0.1594
	hsa03410: Base excision repair	2	18.18	0.0378	*POLD4, PARP3*	9	33	46.32	0.2030	0.1605
	hsa05219: Bladder cancer	2	18.18	0.0467	*CDKN1A, MDM2*	9	41	37.28	0.2232	0.1765
	hsa04151: PI3K-Akt signaling pathway	3	27.27	0.0575	*CDKN1A, MDM2, BCL2L1*	9	345	6.65	0.2471	0.1954
	hsa05200: Pathways in cancer	3	27.27	0.0725	*CDKN1A, MDM2, BCL2L1*	9	393	5.83	0.2622	0.2073
	hsa05214: Glioma	2	18.18	0.0732	*CDKN1A, MDM2*	9	65	23.52	0.2622	0.2073
	hsa05218: Melanoma	2	18.18	0.0797	*CDKN1A, MDM2*	9	71	21.53	0.2636	0.2084
	hsa05215: Prostate cancer	2	18.18	0.0979	*CDKN1A, MDM2*	9	88	17.37	0.3008	0.2378
Downregulation	**hsa03410: Base excision repair**	**4**	**20**	**6.53E-05**	***PCNA, PARP1, NTHL1, APEX1***	**18**	**33**	**46.32**	**0.0026**	**0.0024**
	**hsa04110: Cell cycle**	**5**	**25**	**2.00E-04**	***RB1, CCNB2, CDKN2C, PCNA, SKP2***	**18**	**124**	**15.41**	**0.0036**	**0.0034**
	**hsa03460: Fanconi anemia pathway**	**4**	**20**	**2.72E-04**	***RMI2, RAD51C, UBE2T, RPA1***	**18**	**53**	**28.84**	**0.0036**	**0.0034**
	**hsa05222: Small cell lung cancer**	**4**	**20**	**0.0011**	***RB1, PTEN, CKS2, SKP2***	**18**	**85**	**17.98**	**0.0109**	**0.0101**
	**hsa03420: Nucleotide excision repair**	**3**	**15**	**0.0058**	***PCNA, RPA1, RAD23A***	**18**	**47**	**24.39**	**0.0466**	**0.0431**
	hsa04068: FoxO signaling pathway	3	15	0.0423	*CCNB2, PTEN, SKP2*	18	134	8.56	0.2770	0.2563
	hsa05161: Hepatitis B	3	15	0.0488	*RB1, PCNA, PTEN*	18	145	7.91	0.2770	0.2563
	hsa03430: Mismatch repair	2	10	0.0554	*PCNA, RPA1*	18	23	33.23	0.2770	0.2563
	hsa05200: Pathways in cancer	4	20	0.0693	*RB1, PTEN, CKS2, SKP2*	18	393	3.89	0.2775	0.2567
	hsa03440: Homologous recombination	2	10	0.0694	*RAD51C, RPA1*	18	29	26.36	0.2775	0.2567
	hsa03030: DNA replication	2	10	0.0854	*PCNA, RPA1*	18	36	21.23	0.3107	0.2874

* The analysis was performed using an online tool for functional annotation (DAVID Bioinformatics Resources 6.8). KEGG PATHWAY was used as the reference database.
